# The molecular pathways leading to GABA and lactic acid accumulation in florets of organic broccoli rabe (*Brassica rapa* subsp. *sylvestris*) stored as fresh or as minimally processed product

**DOI:** 10.1093/hr/uhae274

**Published:** 2024-09-28

**Authors:** Giulio Testone, Anatoly Petrovich Sobolev, Maya Dimova Lambreva, Zeineb Aturki, Giovanni Mele, Michele Lamprillo, Francesco Magnanimi, Giovanna Serino, Giuseppe Arnesi, Donato Giannino

**Affiliations:** Institute for Biological Systems, National Research Council (CNR), Via Salaria Km 29,300, 00015 Monterotondo, Rome, Italy; Institute for Biological Systems, National Research Council (CNR), Via Salaria Km 29,300, 00015 Monterotondo, Rome, Italy; Institute for Biological Systems, National Research Council (CNR), Via Salaria Km 29,300, 00015 Monterotondo, Rome, Italy; Institute for Biological Systems, National Research Council (CNR), Via Salaria Km 29,300, 00015 Monterotondo, Rome, Italy; Institute for Biological Systems, National Research Council (CNR), Via Salaria Km 29,300, 00015 Monterotondo, Rome, Italy; Institute for Biological Systems, National Research Council (CNR), Via Salaria Km 29,300, 00015 Monterotondo, Rome, Italy; Department of Biology and Biotechnology, Sapienza Università di Roma, 00185 Rome, Italy; Department of Biology and Biotechnology, Sapienza Università di Roma, 00185 Rome, Italy; Enza Zaden Italia, Strada Statale Aurelia km. 96.400, 01016 Tarquinia, Viterbo, Italy; Institute for Biological Systems, National Research Council (CNR), Via Salaria Km 29,300, 00015 Monterotondo, Rome, Italy

## Abstract

In the context of organic farming, the introduction of a local product to wider markets and an evaluation of storage effects, metabolic and transcriptomic variations in two broccoli rabe genotypes from production cycles of two different years were studied by comparing florets of stored fresh (SF) and packaged (P) for 4 days with those harvested fresh from the field (H). Twenty-five hydrosoluble compounds, including amino acids, carbohydrates, and organic acids, were quantified by untargeted nuclear magnetic resonance (NMR). Principal component analysis produced a neat separation among the three commodity statuses with P being the most divergent and SF closer to H. In the packaged florets, carbohydrate levels dropped significantly (over −52%), while the levels of amino acids and organic acids varied. There was an increase in stress-responsive phenylalanine and valine (over 30%) and succinic and α-ketoglutaric acids (over 75%). Compound correlation analyses indicated a carbohydrate sink towards γ-aminobutyric acid (GABA) and lactic acid (LA) metabolism under hypoxic conditions in packaged florets. RNA-seq analysis revealed that over 4000 genes were differentially expressed in SF vs H and 8000 in P vs H. Several CAR and AA pathways were significantly enriched in S and even more significantly in P, when compared to H. A map of gene expression (175 genes) and metabolite contents (14 compounds) was constructed to elucidate the gene routes that lead to accumulation of GABA and LA, known for healthy properties, in P. WGCNA and promoter binding site analyses enabled the identification of transcription factors (bZIP, WRKY, ERF types), interactions, and targeted genes encoding key enzymes in GABA and LA accumulation.

## Introduction

The species *Brassica rapa* includes important crops, and broccoli rabe represents a culturally significant food in central-southern Italy, known as *cime di rapa* [1] or *friarielli* [2]. It is also known worldwide by various names (broccoli de rabe, raab, rapini, spring broccoli, Italian turnip) due to migratory flows from its domestication areas in Western Asia and the Eastern Mediterranean. The botanical classification of this species is complex; some working groups (www.ecpgr.cgiar.org/) mostly use *B. rapa* L. subsp. *sylvestris* (L.) Janch var. *esculenta* Hort. Since *B. rapa* subsp. *sylvestris* and *B. rapa* are synonyms, another classification system (www.ncbi.nlm.nih.gov/Taxonomy/) creates the ‘Broccoletto group’ (broccoli rabe) within *B. rapa* (L.) subsp. *rapa* for populations intended for shoot production, and the variety *rapa* for those intended for turnips. Analyses based on allele variability split leafy from turnip accessions [3], and the increasing availability of *B. rapa* genome sequences will improve classifications. Genetically, *B. rapa* is diploid (2*n* = 20) and predominantly allogamous with a genome of 425 Mb [4]. Seed selection and multiplication are managed by small companies that produce local varieties (landraces) differing in bolting/harvesting times (40–90–120 days). Large companies minimally invest in breeding programmes (for F1 hybrids), resulting in no cultivars currently listed in the Italian/European registers. However, germplasm banks exist, supported by projects that focus on geo-stratification, unique barcoding of accessions, and SSR marker fingerprinting for breeding [5].

The edible product consists of leaves, stems, and inflorescences (corymbs or florets), the latter being the most appreciated when green with a pungent and slightly bitter taste [1]. It is produced in both conventional and organic farming systems [6] due to the low input needs and the availability of hardy and stress-tolerant landraces, mostly cultivated outdoor in fall to early spring cycles. Broccoli rabe has spread from local to large retail markets, gaining value as a minimally processed product (fresh-cut), and it is rich in compounds with healthy and sensory properties [7, 8], including dietary fibers and glycemic sugars [9], phenolics such as chlorogenic and ferulic acids, flavonols (quercetin, kaempferol, isorhamnetin), tocopherols, ascorbate, and glucosinolates [6, 10, 11]. The latter, responsible for the pungent flavour, may turn useful for landrace fingerprinting [2]. Nutrient contents vary with genotype and tissue [2, 9], season and cultivation systems [6, 7, 12, 13]. Studies on product quality range from fertilization at pre-harvest [12], to air flow composition (3% O_2_, 4°C) during post-harvest storage [11], to optimal gas balance for modified atmosphere packaging (MAP) and film types to limit spoilage during shelf-life [14]. Nutrients and antioxidant capacity in active MAP from a real industrial production chain were affected by processing and storage time [9]. Both packaging conditions and storage duration raise gas concentration inside bags, causing an accumulation of volatile compounds that affect sensory quality. Recently, combining active MAP (3% O_2_ and 97% N_2_) with microperforated polypropylene/polyamide film was shown to be optimal to contain bad odours and preserve organoleptic quality [14].

The variation in amino acid (AA) content and compounds caused by metabolism after product processing and storage has been investigated to a limited extent in broccoli rabe. A recent meta-analysis on foods highlighted the importance of AAs, identified correlations between intake of specific AAs and human diseases, and provided new dietary guidelines [15]. Moreover, the nonprotein γ-aminobutyric acid (GABA), a known neurotransmitter, is reported to have positive effects on human blood pressure and relaxation though these benefits are debated [16]. In plants, GABA interfaces carbon and nitrogen metabolism and acts a signal molecule involved in stress responses [17]. Several abiotic stresses cause GABA accumulation in crop organs [18]. Synthesis occurs via glutamate decarboxylation (GAD; EC 4.1.l.15) in the GABA shunt route, aldehyde dehydrogenation (ALDH, EC 1.2.1.3) following the spermidine degradation, or proline oxidation, while catabolism is achieved by transamination (GABA-T; EC 2.6.1.19) and transport by various carriers [19]. Post-harvest treatments and storage conditions can increase GABA content in brassicaceous vegetables [20], especially under low oxygen conditions. Another by-product under O_2_ limitation is lactate (LA), which plays several roles in diets and human health [21]. Plants convert pyruvate into L-LA stereoisomer [22] by LA dehydrogenases (LDH, EC 1.1.1.27), active at different levels in diverse crops such as lettuce, brassica, and garlic [23] with induction responses to hypoxia [24].

In the context of EU policies tailored to organic farming, an ever-growing organic market [25], and solid consumer preference for organic products in Italy [26], the main objective of this work was to elucidate the variation in the content of selected nutritive compounds in florets—an essential product component—grown under an organic farming system, where packaging is cautiously accepted to maintain the unaltered quality of healthy and pesticide-free production. Low-impact MAP methods may be accepted by organic consumers, but improper MAP can alter organoleptic traits due to accumulation of unpleasant volatiles, as known for rocket and broccoli rabe [14, 27]. Here, the organic product was either stored fresh and ready for consumption or packaged in a real industrial context under minimal perturbation conditions. Metabolic changes and gene expression profiles were evaluated in florets at the likely time of consumption. This study highlights the effects of passive MAP on specimen nutrient levels, indicating that specific bioactive compounds, like GABA and LA, accumulate in bagged products. NMR metabolic profiling and RNA-seq analyses revealed a gene-metabolite network related to these differences, serving as a resource and tool in stress tolerance management and quality improvement.

## Results and discussion

### Variation of nutrient content in florets

**Figure 1:**
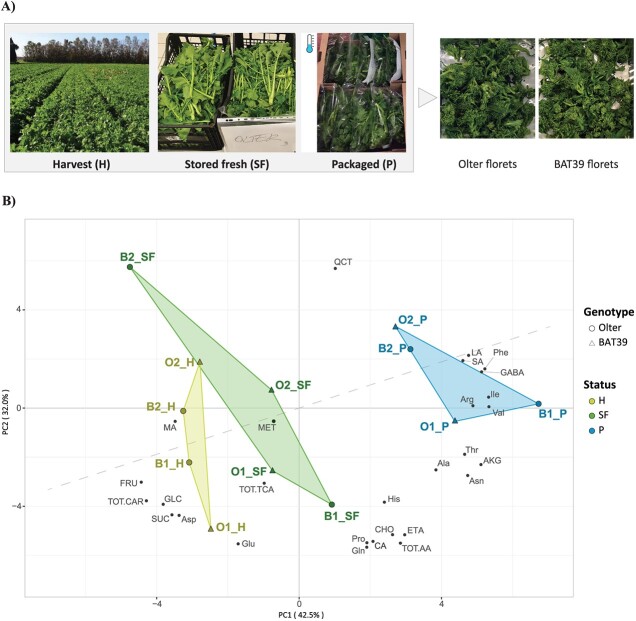
(**A**) Production stages of sampling and type of sample analyzed. Shoots of the Olter and BAT39 (O and B) genotypes were collected at harvest (H), stored fresh as is (SF) 4 days after harvest (4°C), and stored as bagged (P) 4 days after packaging (4°C). Florets (last panel) from the H, SF, and P conditions underwent metabolic and transcriptomic analyses. (**B**) The PCA biplot shows the distribution of 25 hydro-soluble compounds in florets from H, SF, and P conditions of O and B genotypes grown in years 1 and 2 (2021 and 2022). Amino acids: Ile, isoleucine; Val, valine; Thr, threonine; Ala, alanine; Arg, arginine; Pro, proline; Gln, glutamine; Glu, glutamic acid; Asp, aspartic acid; Asn, asparagine; Phe, phenylalanine; His, histidine; GABA, gamma-aminobutyric acid; TOT.AA, total amino acids. Tricarboxylic acids: SA, succinic acid; CA, citric acid; MA, malic acid; AKG, a-ketoglutaric acid; TOT.TCA, total tricarboxylic acids; carbohydrates: GLC, glucose; FRU, fructose; SUC, sucrose; TOT.CAR, total carbohydrates. Other compounds: LA, lactic acid; QCT, quercitin; ETA, ethanolamine; CHO, Choline; MET, methiin.

Twenty-five hydrosoluble compounds from florets (Fig. 1A), assigned by NMR spectra (Table S4), were quantified (Tables 1 and S5). Among these, 20 were categorized by chemical similarity into three groups: carbohydrates, tricarboxylic acids, and amino acids, as listed in Fig. 1. Principal component analysis (PCA) provided a dataset comprehensive view (Fig. 1B) considering the factors genotype (G; BAT39 and Olter), floret commodity status (S), at harvest (H, light green), stored fresh as is (SF, dark green), stored as packaged (P, blue), and production year (Y1, Y2). PC1 and 2 explained 42.5% and 32.0% of the total variance, respectively, with the best separation for P compared to the neighbouring H and SF statuses. ANOVA results indicated that the genotype exerted a lesser influence on metabolite content than the production year and florets status (Table 1). Specifically, genotype significantly (*P* ≤ 0.01) affected 14 out of the 25 metabolites, production year significantly affected 24 out of 25 metabolites, and status affected all metabolites (consistent with PCA findings). Significant interactions were observed for S × Y, G × S and G × Y involving 22, 20, and 14 metabolites out of 25, respectively, underscoring the substantial impact of floret status on metabolite variations. The triple interaction G × S × Y exhibited a highly complex effect requiring nuanced interpretation. Additionally, metabolic profiles of H, SF, and P leaves (Tables S6 and S7) were analysed, revealing significant effects (p ≤ 0.01) of organ type, such as higher levels of total amino acids in florets compared to leaves, and vice versa for tricarboxylic acids (TCA) and carbohydrates (CAR), independent of other variability factors (Table S6).

**Table 1:** S (mg/g DW) in florets of two ‘Cima di rapa novantina’ cultivars from two growth cycles and significances from three-way ANOVA

**Year**	Year1						Year2												
**Genotype**	BAT39			Olter			BAT39			Olter			*Significance*						
**Status**	H	SF	P	H	SF	P	H	SF	P	H	SF	P	**G**	**S**	**Y**	**GxS**	**GxY**	**SxY**	**GxSxY**
Ile	0.13 ± 0.02	0.39 ± 0.02	0.65 ± 0.02	0.18 ± 0.01	0.28 ± 0.02	0.55 ± 0.02	0.16 ± 0.01	0.20 ± 0.01	0.47 ± 0.02	0.11 ± 0.01	0.32 ± 0.01	0.38 ± 0.02	***	***	***	***	***	***	***
Val	0.29 ± 0.04	0.85 ± 0.04	1.42 ± 0.11	0.39 ± 0.01	0.66 ± 0.06	1.29 ± 0.09	0.40 ± 0.03	0.39 ± 0.01	0.94 ± 0.05	0.27 ± 0.02	0.70 ± 0.01	0.80 ± 0.06	n.s.	***	***	***	*	***	***
Thr	0.41 ± 0.07	0.71 ± 0.03	0.84 ± 0.01	0.52 ± 0.02	0.51 ± 0.03	0.74 ± 0.00	0.27 ± 0.02	0.37 ± 0.01	0.58 ± 0.02	0.20 ± 0.01	0.37 ± 0.00	0.53 ± 0.02	***	***	***	***	n.s.	n.s.	***
Ala	0.82 ± 0.13	1.34 ± 0.07	1.98 ± 0.08	0.94 ± 0.07	1.04 ± 0.05	1.16 ± 0.05	1.31 ± 0.01	0.29 ± 0.00	1.56 ± 0.08	0.30 ± 0.01	0.39 ± 0.01	0.86 ± 0.06	***	***	***	***	***	***	***
Arg	0.96 ± 0.07	1.82 ± 0.05	2.59 ± 0.23	0.83 ± 0.04	1.32 ± 0.14	1.81 ± 0.02	0.72 ± 0.10	0.80 ± 0.01	1.46 ± 0.11	1.37 ± 0.49	1.70 ± 0.07	1.69 ± 0.11	n.s.	***	***	**	***	***	n.s.
Pro	4.61 ± 0.36	4.83 ± 0.32	4.51 ± 0.46	4.99 ± 0.52	3.95 ± 0.29	4.14 ± 0.16	1.90 ± 0.19	1.12 ± 0.10	2.25 ± 0.12	1.49 ± 0.19	1.54 ± 0.03	1.63 ± 0.13	*	**	***	n.s.	n.s.	**	***
Gln	13.46 ± 2.55	20.34 ± 0.25	13.92 ± 1.21	17.32 ± 1.34	13.15 ± 0.91	13.59 ± 0.92	13.11 ± 1.27	2.99 ± 0.21	9.92 ± 0.71	9.63 ± 0.29	9.54 ± 0.13	9.55 ± 1.21	n.s.	***	***	n.s.	**	***	***
Glu	6.49 ± 0.98	8.60 ± 0.54	2.86 ± 0.31	7.56 ± 0.44	7.53 ± 0.67	3.64 ± 0.05	3.61 ± 0.29	2.36 ± 0.06	0.78 ± 0.05	2.22 ± 0.23	3.23 ± 0.10	0.99 ± 0.03	n.s.	***	***	n.s.	n.s.	***	***
Asp	3.08 ± 0.41	4.96 ± 0.25	0.70 ± 0.01	3.75 ± 0.32	4.31 ± 0.41	1.06 ± 0.04	2.06 ± 0.22	1.92 ± 0.04	0.29 ± 0.01	2.03 ± 0.05	1.81 ± 0.05	0.52 ± 0.02	n.s.	***	***	***	n.s.	***	**
Asn	1.18 ± 0.21	2.44 ± 0.02	3.18 ± 0.11	1.78 ± 0.14	1.84 ± 0.17	2.77 ± 0.18	1.36 ± 0.13	0.59 ± 0.02	1.82 ± 0.06	1.20 ± 0.08	2.23 ± 0.01	1.85 ± 0.17	***	***	***	***	***	***	***
Phe	0.06 ± 0.01	0.26 ± 0.01	0.68 ± 0.01	0.07 ± 0.01	0.16 ± 0.01	0.57 ± 0.03	0.07 ± 0.01	0.19 ± 0.01	0.45 ± 0.03	0.05 ± 0.01	0.25 ± 0.00	0.38 ± 0.01	***	***	***	***	***	***	***
His	0.69 ± 0.11	0.77 ± 0.07	0.69 ± 0.03	0.72 ± 0.02	0.76 ± 0.04	0.70 ± 0.04	0.64 ± 0.08	0.13 ± 0.02	0.71 ± 0.09	0.73 ± 0.03	0.76 ± 0.06	0.74 ± 0.07	***	***	***	***	***	***	***
GABA	0.23 ± 0.02	0.24 ± 0.03	8.11 ± 0.40	0.23 ± 0.04	0.21 ± 0.03	5.58 ± 0.10	0.13 ± 0.01	0.15 ± 0.01	5.14 ± 0.32	0.09 ± 0.01	0.18 ± 0.01	2.78 ± 0.17	***	***	***	***	n.s.	***	n.s.
**TOT.AA**	**32.41 ± 4.65**	**47.55 ± 1.57**	**42.13 ± 2.77**	**39.26 ± 2.59**	**35.73 ± 2.47**	**37.59 ± 1.11**	**25.73 ± 2.32**	**11.52 ± 0.14**	**26.37 ± 0.54**	**19.69 ± 0.61**	**23.02 ± 0.36**	**22.69 ± 1.68**	n.s.	**	***	*	*	***	***
SA	0.12 ± 0.02	0.05 ± 0.00	0.91 ± 0.05	0.19 ± 0.01	0.04 ± 0.01	0.82 ± 0.02	0.10 ± 0.01	0.20 ± 0.02	0.53 ± 0.03	0.10 ± 0.00	0.03 ± 0.00	0.68 ± 0.04	n.s.	***	***	***	n.s.	***	***
CA	7.82 ± 1.11	12.87 ± 0.89	9.80 ± 0.45	11.05 ± 0.85	11.66 ± 0.52	10.56 ± 0.32	6.03 ± 0.55	5.10 ± 0.14	6.90 ± 0.60	5.71 ± 0.30	7.36 ± 0.20	5.76 ± 0.40	**	***	***	**	n.s.	***	***
MA	5.19 ± 0.68	3.97 ± 0.23	2.93 ± 0.10	6.73 ± 0.59	3.05 ± 0.36	2.98 ± 0.18	3.86 ± 0.43	12.31 ± 1.16	2.28 ± 0.35	2.81 ± 0.21	1.90 ± 0.10	1.67 ± 0.20	***	***	n.s.	***	***	***	***
AKG	0.49 ± 0.08	0.92 ± 0.10	1.33 ± 0.06	0.65 ± 0.07	0.74 ± 0.09	1.15 ± 0.01	0.29 ± 0.03	0.17 ± 0.01	0.80 ± 0.05	0.34 ± 0.05	0.72 ± 0.03	0.75 ± 0.02	**	***	***	***	***	**	***
**TOT.TCA**	**13.61 ± 1.85**	**17.81 ± 0.74**	**14.97 ± 0.58**	**18.63 ± 1.4**	**15.5 ± 0.96**	**15.51 ± 0.44**	**10.28 ± 1.00**	**17.79 ± 1.13**	**10.51 ± 0.98**	**8.94 ± 0.43**	**10.00 ± 0.31**	**8.87 ± 0.63**	***	***	***	***	***	***	**n.s.**
GLC	6.95 ± 0.18	6.03 ± 0.35	1.79 ± 0.21	12.61 ± 2.22	5.92 ± 0.77	4.44 ± 1.16	8.05 ± 1.18	5.02 ± 1.08	3.57 ± 0.48	4.04 ± 0.58	3.97 ± 0.61	1.96 ± 0.29	n.s.	***	***	n.s.	***	***	***
FRU	11.53 ± 0.51	7.51 ± 0.33	2.57 ± 0.12	13.67 ± 1.67	6.35 ± 0.42	2.77 ± 0.48	9.49 ± 1.17	8.05 ± 1.55	4.26 ± 0.41	4.28 ± 0.24	5.94 ± 0.44	2.31 ± 0.20	***	***	***	n.s.	***	***	***
SUC	13.56 ± 0.96	5.62 ± 0.21	0.81 ± 0.08	13.24 ± 0.63	4.97 ± 0.34	1.37 ± 0.22	7.22 ± 0.60	1.40 ± 0.20	1.64 ± 0.14	3.90 ± 0.35	6.52 ± 0.21	1.64 ± 0.07	n.s.	***	***	***	*	***	***
**TOT.CAR**	**32.03 ± 1.52**	**19.17 ± 0.69**	**5.17 ± 0.29**	**39.52 ± 4.05**	**17.23 ± 0.88**	**8.58 ± 1.2**	**24.77 ± 2.95**	**14.48 ± 2.77**	**9.47 ± 1.00**	**12.23 ± 1.16**	**16.43 ± 1.19**	**5.91 ± 0.55**	n.s.	***	***	n.s.	***	***	***
LA	0.00 ± 0.00	0.00 ± 0.00	1.55 ± 0.05	0.00 ± 0.00	0.00 ± 0.00	0.78 ± 0.05	0.01 ± 0.00	0.03 ± 0.00	1.89 ± 0.12	0.01 ± 0.00	0.01 ± 0.00	0.86 ± 0.07	***	***	***	***	**	***	**
QCT	0.28 ± 0.02	0.35 ± 0.06	0.81 ± 0.05	0.16 ± 0.03	0.16 ± 0.01	0.42 ± 0.03	0.54 ± 0.05	1.36 ± 0.06	1.35 ± 0.11	0.58 ± 0.04	0.56 ± 0.03	0.78 ± 0.14	***	***	***	***	***	***	***
ETA	0.61 ± 0.09	0.77 ± 0.04	0.78 ± 0.03	0.78 ± 0.06	0.64 ± 0.05	0.71 ± 0.01	0.63 ± 0.05	0.27 ± 0.01	0.64 ± 0.02	0.53 ± 0.08	0.56 ± 0.01	0.53 ± 0.02	n.s.	***	***	***	n.s.	***	***
CHO	1.99 ± 0.20	2.35 ± 0.22	2.44 ± 0.16	2.51 ± 0.16	2.07 ± 0.07	2.32 ± 0.13	2.06 ± 0.18	0.82 ± 0.06	1.97 ± 0.11	1.84 ± 0.13	1.83 ± 0.03	1.64 ± 0.11	n.s.	***	***	***	n.s.	***	***
MET	3.02 ± 0.19	1.77 ± 0.08	2.45 ± 0.08	3.92 ± 0.21	4.96 ± 0.06	3.89 ± 0.17	4.92 ± 0.35	1.77 ± 0.08	3.96 ± 0.10	5.42 ± 0.18	4.96 ± 0.06	3.47 ± 0.21	***	***	***	***	***	***	***

Abbreviation list is in the legend of Fig. 1B. G, genotype; S, status; Y, production year. ns, nonsignificant; *, **, *** = significant at *P* ≤ 0.05, 0.01, and 0.001, respectively.

The discussion of metabolite contents primarily quotes literature on *Brassica* spp. florets, analysed via NMR or other specified techniques, with concentrations converted based on cited data. Moreover, the following text focuses on content variations addressing only fresh-cut florets under passive MAP at 4 days post-packaging (DPP), which is the presumed optimal consumption time with a 7-day sell-by-date, compared to freshly harvested florets. At 4 DPP, the bags contained average oxygen and carbon dioxide levels of 10% and 12%, respectively, differing from the initial packaging stage where oxygen was 21% and carbon dioxide was 0.03% (Table S8). Pigment content (chlorophyll and carotenoids, Table S9) remained consistent with that observed at 1 DPP, with no visible signs of tissue yellowing (chlorosis) or floret opening. In contrast, improper MAP methods for broccoli rabe lead to spoilage due to undesirable odours and discoloration [14]. The gas conditions of this work (p-MAP in anti-fog polypropylene at 4 DPP) were comparable to those of broccoli rabe shrink-wrapped in plastic cling film at 5 DPP and in active MAP with porous film at 12 DPP, with this latter condition being indicated as the best optimal choice for maintaining organoleptic properties [14]. Notably, at 7 DPP, the CO_2_ content increased further (over 70% vs 4 DPP), suggesting a potential increase in volatile compounds (although not addressed in this study), as indicated by the upregulation of acetaldehyde synthesis genes at 4 DPP (see RNA-seq section). Hence, at the conditions of the present industrial process, organic broccoli rabe consumption should be no later than 4 DPP. Variations in the content of selected classes of compounds are described below.

### Amino acids

The total amino acids (TOT.AA) range was 11.52–47.55 mg/g DW (Table 1). Significant influences of commodity status and production year was significative on all AAs, while genotype affected 8 out of 13 AAs. The S × Y, G × S, G × Y interactions affected 12, 10, and 7 AAs, respectively, confirming the higher incidence of floret status. Finally, the G × S × Y interaction affected 11 out of 13 AAs. Comparing packaged products with freshly harvested ones (Table S5), TOT.AA relative variation content increased from −4 to +30% in Y1 and from +2 to +15% in Y2, with varying contributions of each AA between genotypes. Remarkably, Ile, Val, Thr, Ala, Arg, Asn, Phe, and GABA showed significant increases (e.g., Phe over 550%, GABA over 2300%) in the packaged product regardless of year or genotype. Conversely, Glu and Asp levels decreased by more than 50% in both years and genotypes, while Gln content declined in bagged florets of Olter in Y1 (−22%) and BAT39 in Y2 (−24%) or showed modest variations (±10%). The TOT.AA levels reported here were significantly higher (7–20-fold) than those reported in turnip tops of *Brassica rapa var. rapa* [28]. Differences in analytical techniques (NMR vs HPLC), AA panels (13 vs 16), or sample composition (florets vs a mixture of leaflets, stem, and florets) may explain these discrepancies. Studies on broccoli (*Brassica oleracea* var *italica*), using both HPLC [29] and NMR [30] methods, report ranges (4.5 and 8.8 g/kg FW) closer to the values of this work (2.8–5.7 g/kg FW, here converted using the DW/FW for florets of 14.6%). In white cauliflower (*Brassica oleracea var. botrytis*), AA content in florets (based on 15 AAs via NMR) averaged 76.5 mg/g DW [31], consistent with findings here. In all the above works, Glu, Gln Asn, Asp, Arg, and Phe recurred as the most abundant in the AA content, also observed here. Essential AAs such as Phe and Val showed relative increases of 550–1133% and 134–390%, respectively, in packaged products (Table S5), which is beneficial considering their role in diets combating obesity [15]. Among non-essential AAs, GABA contents in various *Brassica* spp. were reviewed. Raw broccoli florets (*B. oleracea*) and green mustard flower buds (*B. juncea*) showed similar ranges [32], with GABA level of 31–40 mg/kg FW for broccoli and 13–33 mg/kg FW for freshly harvested broccoli raab florets. In *B. oleracea*, GABA content significantly increased after 7 days of storage under enriched CO_2_ atmosphere [20], while in broccoli florets AA levels—including GABA—strongly decreased after bottling and freezing following blanching [29]. The microoxic conditions during processing, potentially triggering GABA accumulation (not tested in this study), and subsequent shelf-life conditions leading to CO_2_ accumulation in bags, align with the accumulation patterns observed for other microoxic-responsive AAs such as Ala, Val, Ile, and Glu [33].

### Carbohydrates and organic acids of the TCA cycle

The total carbohydrates (TOT.CAR) range was 5.17–39.52 mg/g DW (Table 1). The G influence was limited to fructose (FRU), while the S and Y effects were significant for all sugars. For example, BAT39 and Olter had a similar sugar content (32.03 *vs* 39.52 mg/g DW) at harvest in Y1, while it decreased in Y2, but was twofold higher in BAT39 than in Olter (24.77 vs 12.23 mg/g DW). Overall, the packaged product exhibited an 84% and 78% decrease in TOT.CAR content in Y1, and a 62% and 52% decrease in Y2 for BAT39 and Olter, respectively (Table S5). This decrease was primarily due to reductions in glucose (GLC), fructose (FRU), and sucrose (SUC), with SUC showing the most pronounced decrease (over 90% in Y1 and 58% in Y2 for both genotypes). In a previous work [9], total carbohydrate contents (SUC, FRU, GLU) determined by HPLC in the entire shoot of broccoli rabe ranged from 2.2 to 7.0 g/100 g DW, much lower than the values found here specifically for florets (122–395 g/100 g DW), while less divergent amounts were quantified [34] in broccoli by NMR as 8.5–13.4 mg/g DW (here 1.8–5.8 mg/g DW).

The sum of succinic acid (SA), citric acid (CA), malic acid (MA), and α-ketoglutaric acid (AKG) contents varied from 8.87 to 18.63 mg/g DW with CA (5.10–12.87 mg/g DW) and MA (1.7–12.9 mg/g DW) as the most abundant, while AKG and SA ranged from 0.03 to 1.33 mg/g DW. Product status significantly affected all four TCAs, both independently and in interactions (Table 1). For packaged florets (Table S5), SA and AKG contents showed remarkable increases (from +329% to +664% and from +76% to +179%, respectively) compared to freshly harvested products, while MA levels decreased by more than 40%, irrespective of cultivar and year; CA exhibited either incremental (+25% in BAT93/Y1 and + 14% in Olter/Y2) or essentially unchanged trends. In plant TCA cycles, MA and CA are typically the most abundant, consistent with the ranges observed in turnip tops (37.12 ± 12.41 and 101.42 ± 38.72 mg/100 g FW [35], which align with measurements in harvested florets (41.0–98.3 and 83.4–161.3 mg/100 g FW). Specific studies on fruit responses to hypoxia have highlighted the accumulation of SA and AKG (and lactate) but not CA, indicating a downregulated TCA cycle halting at SA and initiating the GABA shunt [36].

### Other compounds: lactate, quercetin derivative, ethanolamine, choline, and methiin

The lactate (LA) content ranged from undetectable levels (below the NMR threshold) to 0.01–0.03 mg/g DW in both harvested and fresh-stored florets (Table 1). Conversely, higher values (0.78–1.86 mg/g DW) occurred in packaged florets, suggesting floret-originated LA accumulation. This is supported by disinfection during washing, absence of microbial reads in RNA-seq data, and upregulation of *LDH* genes, indicating its role in hypoxic metabolism of aerial organs [23, 24].

Quercetin (QCT) content ranged from 0.16 to 1.36 mg/g DW and was significantly influenced by all factors and their interactions (Table 1). Packaged florets showed higher contents (+34% to +186%) compared to harvested ones regardless of cultivar and production year (Table S5). In contrast, turnip tops of *B. campestris* contained much lower levels (7.3 mg/kg FW) [37] than reported here (23.4–84.7 mg/kg FW). Flavonol levels typically drop in packaged broccoli rabe during storage, suggesting the reported QCT increase may involve its glycosylated forms. The parallel accumulation of QCT and Phe aligns with their metabolic link in the phenylpropanoid pathway.

ETA and CHO levels were unaffected by G and G × Y interaction but significantly by S and Y and all interactions (Table 1). ETA (0.27–0.78 mg/g DW) and CHO (0.82–2.51 mg/g DW) contents varied between bagged and fresh florets, sometimes in opposite directions, e.g., ETA content in bagged Olter (+27%) *vs* BAT39 (−9%) of Y1 (Table S5). In cabbage (*B. oleracea*) florets, CHO and ETA amounts by NMR ranged between 4.73 and 0.79 mg/g DW [31], in agreements with ranges recorded here (respectively, 1.84–2.51 and 0.53–0.78 mg/g DW).

Methiin (MET) content varied significantly across all factors and their interactions. Negative trends (from −19 to −36%, Table S5) were observed in all bagged versus fresh products (except bagged florets from Olter/Y1, which showed no variation). In broccoli florets, the content at harvest was above 0.5 g/kg FW [38] in agreement with the values (0.44–0.79 g/kg FW) found for the florets in this study. An average of 20.71 mg/g FW was measured in cauliflower by NMR methods, about 5–7 times higher than what we observed in florets (3.02–5.42 mg/g FW). Broccoli storage in low oxygen conditions caused strong off-flavours due to volatile sulphur compound generation resulting from MET decomposition by cystine lyase; the decrease in MET levels observed in fresh or packaged florets may reflect their conversion to S-volatiles. However, this hypothesis was not supported by the gene expression analyses showing a steady state or downregulation of cystine lyase genes in stored florets.

### Correlation analysis

Correlation analysis (Fig. S1) explored relationships among CAR, TCA, and AA to define the physiological status of packaged florets and integrate transcriptomic analyses within the GABA and lactate pathways. Below, we report on significant correlations (r ≥ |0.70|, p ≤ 0.05) and discuss some of them in relation to well-characterized biochemical routes in plants. Briefly, the contents of SUC/GLC/FRU, linked by a mutual positive correlation (r > 0.8), were all negatively correlated with those of lactic and SAs (e.g., r ≤ −0.74 for SUC and FRU); the sugar group was also negatively correlated with GABA, Phe, and Arg (only GLC/FRU *vs* the latter). CA-AKG-SA sequential acids of Krebs cycle were linked by positive correlations and revealed strong correlations (r ≥ |0.70|), exclusive or overlapping, with several AA (e.g., CA had r ≥ 0.72 with Gln, Glu and Pro; AKG had r ≥ 0.73 with Arg, Asn, and Thr, and shared with SA those with GABA, Phe, Val). GABA and LA were highly correlated (r = 0.96) and shared positive correlations (r > 0.76) with SA, and negative correlations (r ≤ −0.74) with Asp. GABA also correlated positively with AKG and Thr (r > 0.72). CAR trends suggest that the sugar carbon is mainly channeled into GABA and LA accumulation by involving SA and Asp. GABA and LA increased levels are typical in plants suffering from hypoxia [39] and are likely to occur in packaged florets (Table S5). The parallel raise of key TCA levels, as well as in Phe and Arg levels might reflect the plant stress status, since the former AA is a precursor of phenolic compounds and the latter of nitric oxide and polyamines, well-known markers of abiotic stress [40, 41].

### Transcriptomic variations in florets

#### Transcriptome sequencing and differential expression analysis

**Figure 2:**
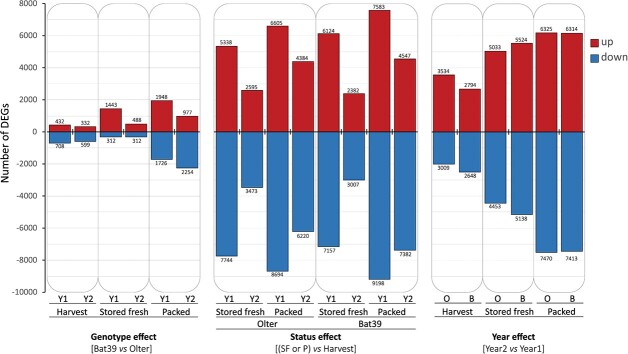
Plot of differentially expressed genes (FDR ≤ 0.05, absolute log_2_ fold change ≥1) due to the genotype effect (differences between BAT39 *vs* Olter scored in each product type), the commodity status (differences within each genotype between conserved products *vs* harvest) and the production year (differences between Y2 vs Y1, in each genotype and status). The number of up- and down-regulated genes is given at the top of the respective bars for each comparison.

Transcriptome variations were monitored in the same samples as those used for metabolite profiling, namely BAT39 and Olter florets at H, SF, and P commodity status from Y1 and Y2 productions. Approximately 2100 million read pairs were generated from sequencing (36 paired-end libraries; average depth ca. 59.6 million reads/sample). Over 97.5% passed quality filtering and adapter removal, with 83.2% aligned to *B. rapa* reference genome. PCA (Fig. S2) explained 84% of the variance, mainly due to PC1 (70%). Genotype, floret status, and production year clustered distinctly, with P products of both genotypes and years in positive PC1 quadrants, separate from the H and SF. Fig. 2 summarizes the number of differentially expressed genes (DEGs) as influenced by the genotype (left panel), product status (middle panel), and production year (right panel. Floret status had a greater effect on the total DEGs number (range: 5389–16 781) than genotype (800–3674), and more like the production year (5442–13 795). The highest DEGs number occurred in bagged BAT39 florets from Y1 (middle panel), of which 9198 (55%) and 7583 (45%) genes were respectively down- and up-regulated (blue and red bars) compared to the harvest status. More down-regulated than up-regulated genes were noted in SF and P products. Comparative omics analyses related to different aspects of postharvest broccoli (*B. oleracea* var *italica*) have provided insights into several pathways [42, 43]. Our work enriches investigation in previously unexplored pathways in florets of Broccoli raab.

#### GABA and lactic acid pathways

**Figure 3:**
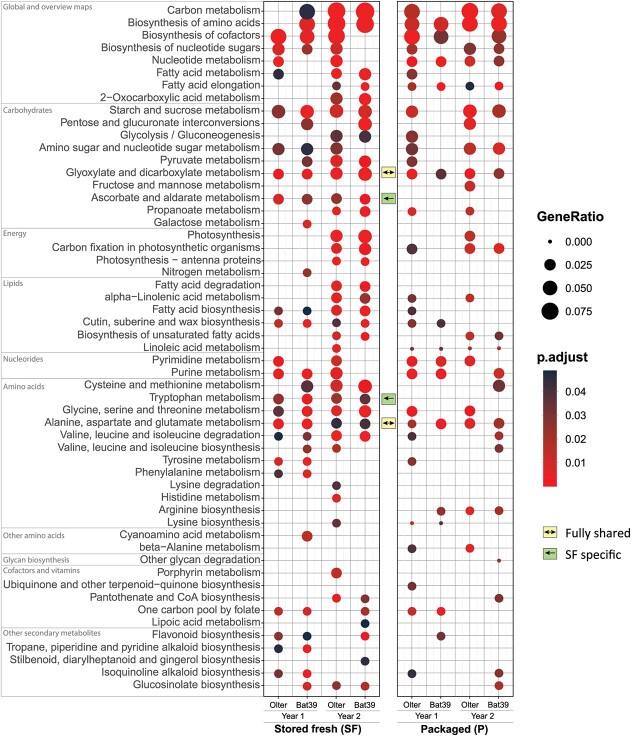
KEGG pathway enrichment analysis. The KEGG maps (y-axis) were grouped into boxes according to their KEGG-Level-2 categories. The dot size represents the gene ratio (number of DEGs within a given pathway over the total number of DEGs) found in each genotype within a given production year (*x*-axis). Enrichments in SF and P products are shown in the left and right panels, respectively. The colour scale represents the Benjamini–Hochberg *p*-adjusted values. Double and single arrows indicate metabolic pathways altered between all conditions (double arrow) or specific to a given condition (single arrow).

KEGG (Figs 3) and GO term (S3) enrichment analyses were performed to explore functional roles of DEGs comparing packaged florets with freshly harvested ones. We focus on KEGG results related to GABA and lactate pathways. Both SF and P fully shared the enrichment of some metabolic pathways regardless of genotype and production years (e.g., ˈglyoxylate and dicarboxylate metabolismˈ and ˈalanine, aspartate and glutamate metabolismˈ marked by bidirectional arrows), or partially shared (7 out of 8 cases, e.g., AA biosynthesis, starch, and sucrose metabolism). While the Arg biosynthesis enrichment characterized most of the P but not the SF florets, the tryptophan and ascorbate metabolisms (green arrows) were specific for the SF product. The contents of these compounds need further investigation that will be addressed in future works.

**Figure 4:**
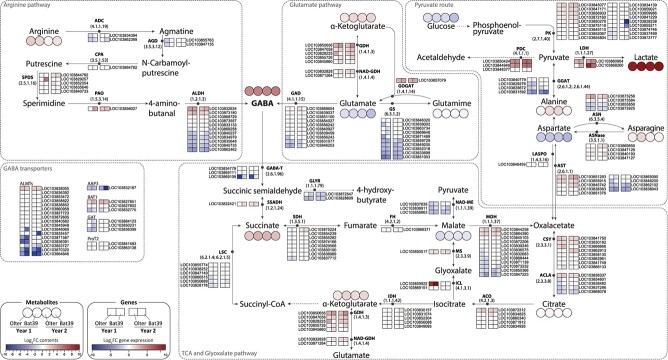
Gene-metabolite map of the GABA and lactic acid pathways in florets from packed cultivars. Differential gene expressions (squares) and differential compound abundances (circles) in Olter and BAT39 cultivars from two production cycles are highlighted by heatmaps according to log2 fold change (log2FC) with respect to harvested products. Arginine pathway: ADC, arginine decarboxylase; AGD, agmatine deiminase; ALDH, aldehyde dehydrogenase (NAD+); CPA, N-carbamoylputrescine amidase; PAO, polyamine oxidase; SPDS, spermidine synthase. Glutamate pathway: GAD, glutamate decarboxylase; GDH, glutamate dehydrogenase [NAD(P)+]; GOGAT, glutamate synthase (NADH); GS, glutamine synthetase; NAD-GDH, glutamate dehydrogenase (NADP+). Pyruvate route: ASN, asparagine synthase; ASNase, asparaginase; AST, aspartate transaminase; GGAT, glutamate—glyoxylate aminotransferase 1; LASPO, L-aspartate oxidase; LDH, L-lactate dehydrogenase; PDC, pyruvate decarboxylase; PK, pyruvate kinase. TCA cycle: ACLA, ATP citrate synthase; ACO, aconitate hydratase; CSY, citrate (Si)-synthase; FH, fumarate hydratase; GABA-T, 4-aminobutyrate—pyruvate transaminase; GLYR, glyoxylate reductase (NADP+); ICL, isocitrate lyase; IDH, isocitrate dehydrogenase; LSC, succinyl-CoA synthetase; MDH, malate dehydrogenase; MS, malate synthase; SDH, succinate dehydrogenase; SSADH, succinate-semialdehyde dehydrogenase (NAD+). GABA transporters: AAP3, amino acid permease 3; ALMTs, aluminium-activated malate transporters; BAT1, bidirectional amino acid transporter 1; GAT1, GABA transporter 1; ProT2, proline transporters 2.

GABA and LA accumulated in packaged florets independently of genotype and production year (Tables 1 and S5). We designed a map of GABA and LA metabolism, illustrating fold change in gene expression and metabolite levels in packaged products compared to freshly harvested products (Fig. 4). DEGs from SF *vs* H conditions are reported in Table S10. In Fig. 4, all genes were selected as the closest and/or at the minimum distance from the quantified compounds. Hereafter, with the aim to provide the most robust results, we report here the metabolic/transcriptomic correlation patterns that recurred in two independent experiments (two production cycles in two different years), each of them based on three biological replicate bulks (in support of representativeness of the material with samples of high similarity, see PCA for metabolites in Fig. 1B and for transcriptomics in Fig. S2). The GABA-related pathways included: the Arg pathway, composed of 22 genes leading to aldehyde dehydrogenase (ALDH, EC: 1.2.1.3); the Glu route, involving 28 genes leading to glutamic acid decarboxylase (GAD, EC: 4.1.1.15); and the TCA cycle harbouring 63 genes that unfolds from the GABA-transaminase (GABA-T, EC: 2.6.1.96). The map also includes the differential gene expression of the major GABA transporters (26 genes). The LA-related pathways encompassed the phosphoenolpyruvate to pyruvate (PEP to Pyr, 18 genes) and the oxalacetate to pyruvate (OAA to Pyr, 19 genes) routes both providing substrate to lactate dehydrogenase (LDH, EC: 1.1.1.27). Below, we mainly report on DEGs that occurred in both genotypes and years to explain the increase in compound contents. At 4 days post-packaging (DPP), GABA content may result from the Arg pathway via up-regulation of some *ALDHs* rather than from GAD-driven synthesis, as *GAD* genes showed unvaried/downregulated expression. The down regulation of the key catabolic *GABA-T* of most GABA transporters (e.g., *ALMTs; AAP3*) supports GABA accumulation, which may trigger *BAT1* mitochondrial transporters. The significant up-regulation of several *pyruvate kinases* (*PK*) genes point at LA synthesis fuelled by the conversion of PEP to Pyr rather than from OAA to Pyr route, as supported by the repression trend of *glutamate:glyoxylate aminotransferase* (*GGAT*) genes. Finally, the gene-metabolite map of GABA-LA metabolism (Fig. 4) suggests that florets accumulate acetaldehyde and glyoxylate, as supported by the up-regulation of *pyruvate decarboxylase* (*PDC), isocitrate liase* (*ICL*), and *malate synthase* (*MS*) genes. This suggests that the packaged florets were undergoing low oxygen conditions. Indeed, the end-by products of the PDC enzyme are acetaldehyde and CO_2_ (using Pyr), and those of the MS and ICL enzymes are glyoxalate (using malate and isocitrate, respectively), which are known to accumulate in plants under hypoxic stress.

Several studies suggest that the expression levels of GABA- and LA-related genes are correlated with their respective protein content and enzymatic activity under stress conditions. For instance, *GABA-T* ectopic expression in mulberry under stress caused GABA decrease, indicating a direct link between gene and enzyme function [44], supporting the relationship observed in this work. As for LDH, hypoxic conditions in maize led to LDH activity increase that correlated with higher mRNA levels of two *LDH* genes [24]. Consistently, the overexpression of *LDH1* gene in Arabidopsis improved survival under low-oxygen stress, supporting direct connections among gene expression, enzyme activity, and stress resilience [45]. Moreover, though post-translational mechanisms cannot be excluded, e.g., those reported for mammalians [39], no evidence has been reported for GABA and LDH-related enzymes in plants, to our knowledge, hence they were not addressed in this study. Nevertheless, we think that the gene and metabolite patterns observed to recur across different experiments under stringent conditions provide robustness for the correlations between gene expression and metabolite levels.

Overall, these results are consistent with changes in carbon and nitrogen metabolism that occur during storage of fresh or fresh-cut leafy vegetables. These changes often involve the accumulation of stress-responsive AAs, derived from both *de novo* synthesis and protein degradation, and necessary to maintain osmotic homeostasis [40]. Secondly, transcriptomic and compound analyses converge to depict the response of florets to microoxic conditions caused by respiration as confirmed by the CO_2_ raise (Table S8). Consistently, the increase in GABA and LA contents is sporadic in the SF product kept in normal atmosphere (GABA increases only in Olter and LA only in in both genotypes for Y2), as supported by the unvaried expression of key pathway genes (Table S10). The GABA metabolic changes in post-harvest commodities have been reviewed [41], showing that even in broccoli there is still work to be done in terms of transcriptomics. In tomato storage under high CO_2_, GABA accumulation resulted from *GABA-T* repression and not from *GAD* induction [41]. Relatedly, the key role of *GABA-T* (degradation) in the high GABA accumulation in packaged florets in this work is also supported by the intense effects on GABA increase when the gene is inactivated in tomato [46]. Moreover, the *GAD* unvarying expression in bagged florets may be due to known autoinhibitory mechanisms [47]. Here, the high GABA content might also reflect the polyamine catabolism, but the complex transcriptional patterns (e.g., the contrasting up- and down-regulation of *ALDH* genes) and not having addressed enzymatic activity did not allow a directional conclusion on the contribution of this pathway to GABA accumulation in packaged florets. Finally, given the role of markers to a set of genes found here, microoxic packaging conditions may prove useful for increasing bioactive compounds. However, the trade-off with organoleptic qualities, which may be affected by the accumulation of volatiles and polyamines, requires careful evaluation.

#### Weighted Correlation Network Analysis (WGCNA) and best candidate transcription factors controlling GABA and LA metabolism

**Figure 5:**
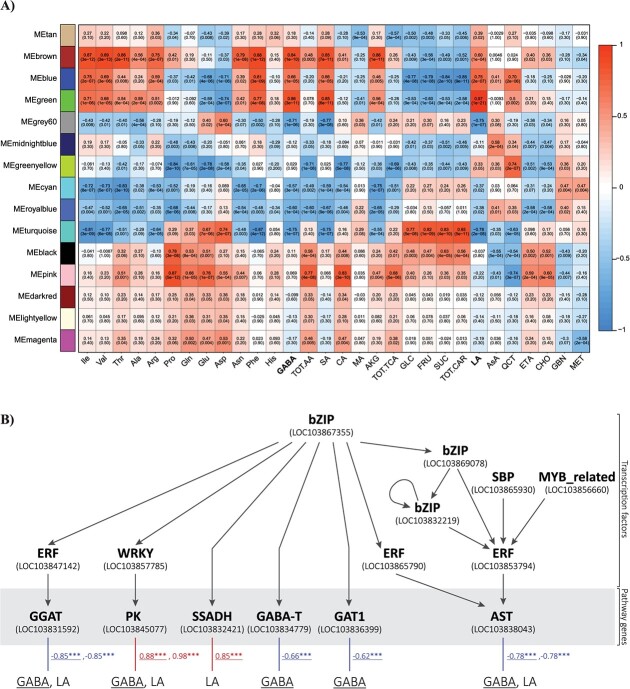
Results of WGCNA and model of regulation of GABA- and LA-related genes. (A) Heatmap of module-metabolite relationship showing the correlation between module eigengenes (*y*-axis) and the quantified compounds (*x*-axis). The numbers in the boxes refer to the Pearson correlation indices and corresponding bracketed *p*-values (“rcorr” function). The strength of the correlation is indicated by a colour gradient as shown in the colour legend. Metabolite abbreviations are given in legend of Fig. 1. (B) Scheme of the proposed regulatory hierarchy of GABA- and LA-related genes. Arrowheads indicate putative direct interaction between TF and target genes based on binding site search; simple lines indicate relationships between pathway genes and metabolites based on positive or negative correlation indices. *** = significant at *P* ≤ 0.05, 0.01, and 0.001, respectively.

WGCNA was carried out to identify groups of coexpressed genes (modules) whose variation correlated with that of the quantified compounds (Fig. 5). Overall, 15 modules consisting of 19–2439 genes were identified (Tables S11); the green, brown, blue, turquoise, and grey60 modules were selected based on the highest correlation (*r* > |0.70|; *P* ≤ 0.05) with GABA and LA fluctuations (Fig. 5A). Specifically, the green module (245 genes) showed positive correlation with the two metabolites (*r* = 0.86 and 0.97, respectively), whilst the turquoise (2439 genes) and the grey60 (69 genes) ones showed negative correlations with the two (*r* = −0.75 and − 0.71 *vs* GABA; *r* = −0.78 and − 0.75 *vs* LA). The brown (856 genes) and the blue (904 genes) modules showed positive correlation specifically with GABA (*r* = 0.84) and LA (*r* = 0.75). A set of genes from the five modules was screened for the highest gene significance, module membership and intra-modular connectivity and used to construct a correlation network that distinguished transcription factors (TFs) from enzyme encoders (Fig. S4). A second round of gene screening, which searched for binding sites in gene promoters targeted by TFs, identified 53 regulations, of which 25 were unique, within the gene set (Table S12), allowing the construction of a putative core network regulating GABA and LA metabolism. (Fig. 5B). Briefly, the bZIP, SBP, MYB_related, ERF, and WRKY class TFs were proposed to act on enzyme-encoders (*PK, GGAT, SSADH, GABA-T,* and *AST*) and transporters (*GAT1*). The bZIP (*LOC103867355*) may act upstream, directly regulating *SSADH* (*LOC103832421*), *GABA-T* (*LOC103834779*), and *GAT1* (*LOC103836399*) and indirectly *GGAT* (*LOC103831592*), *PK* (*LOC103845077*), and *AST* (*LOC103838043*) by interaction with other TFs (WRKY/*LOC103857785*, ERF/*LOC103847142*, and ERF/*LOC103865790*). Moreover, the ERF/*LOC103853794* may act downstream of a multi-TF regulatory circuit (bZIP-SBP-MYB_related) to control *AST* that works between the TCA pathway and Pyr-to-LA branch.

Looking at literature on these TF function based on experimental evidence, WRKY and bZIP factors have roles in modulating GABA contents in plant challenged by abiotic stress [48]. The Arabidopsis homologous of bZIP/LOC103867355 is bZIP16, which has been shown to interact with other G group bZIP factors [49], support a possible interaction in our system. However, the interplay between bZIP and SSADH, GABA-T, and GAT1 is still unveiled in model species. Moreover, DREB (an ERF type) is GABA-responsive in regulating water deficiency stress in clover seeds [50] sustaining the possible role of ERF/LOC108847142 on GGAT. DREB was shown to enhance abiotic stress tolerance in transgenic Arabidopsis and cabbage [51], although we could not find literature data directly linking this TF to GGAT gene regulation. The control of pyruvate kinases by WRKY in kaki [52] and the interaction of Arabidopsis WRKY33 with kinases to regulate GABA-related GAD genes (glutamate decarboxylase) under biotic stress [53] support the role of WRKY/LOC103857785 (best reciprocal hit with WRK33) in controlling PKs involved in GABA and LA pathways. Finally, although AST and ERF are both crucial in plant stress responses, no direct interactions between them have not been reported to our knowledge, bringing up the need for specific research in this area.

## Conclusion

The analysis of metabolic and transcriptomic changes carried out on broccoli rabe florets stored as fresh and packaged commodities in comparison with the harvested product brought to light the variations in several nutrients, and genes subtending their pathways, particularly of amino acids, which showed content variation depending on the different status and preservation. Importantly, the packaged florets accumulated plant g-aminobutyric and lactic acids at the consumption stage due to the down-tuning of GABA catabolism and transport, together with the triggering of Arg catabolism, and the direct synthesis of LA, favored by conditions of increasing anoxia.

## Material and methods

### Plant materials and growth conditions.

The genotypes ‘Cima di rapa novantina’ (Olter company, now Blumen Group, hereafter defined as “Olter”) and the unregistered BAT39 (Enza Zaden Italia) were grown in the open field at the organic farm Biocaramadre (Fiumicino Rome, Italy, 41°53′45” N 12°13′36″ E; 10 m. above mean sea level), in two autumn-winter cycles under Regulation (EU) No. 2018/848; the sowing and harvest dates were: 11 November 2020–16 February 2021 (97 days) and 5 November 2021–7 February 2022 (94 days), respectively. Before sowing, soil was enriched with organic matter by 400 kg/ha of Prodigy Plus (Biogard, www.biogard.it) and with S, Mg, and K by 400 kg/ha of Pantetkali (FertilSud, www.kpluss.com/en-us). Precision mechanized direct seeding was carried out in a ridge planting system, achieving 33 ± 4 plants/m (ca. 200–230 plants/m^2^), and harvest yielded an average of 22 t/ha. Irrigation (water quality and quantity available on request) by springling was carried out if soil tension reached -500 mbar (Tensiometer Art. 8059, Stelzner®). Two treatments (n. 2) against rust (*Albugo candida*) were done at first visible symptoms using 2 kg/ha zeolites (Zeocarb, www.biogard.it/) +1.5 kg/ha Vitobiosat (soluble Cu 5%, Zn 1%; NDGGroup SRL, www.ndggroup.eu). Tables S1 and S2 report parameters on soil, temperature, relative humidity, and precipitation for the two cycles of cultivation.

### Sampling methods and criteria

A production flowchart and an overview of sampling times and sample types are shown in Fig. 1A. Briefly, the product was sampled in three different conditions: at harvest (immediately frozen in liquid nitrogen in field), stored fresh as is (4 days post-harvest in cells kept at dark, 5 ± 1°C, under ventilation and natural air gas composition) and stored as bagged (4 days post-packaging, same conditions as above); here abbreviated as H, SF, P, respectively. The time of sampling was chosen based on an estimate of the highest probability of product consumption by the customer, considering that current legislation imposes consumption within 2 days after bag opening (Regulation EU No 1169/2011 and Italian DM n° 3746-2014) and that stakeholders converged on the maximal sell-by-date at 7 days post packaging (DPP) [54]. Three crates of fresh product (net weight: 4.5 kg/crate) per genotype were used. Florets were manually separated from leaves and stems (borne on the entire shoots) to form three replicate bulks (RB) of florets. As for the processed material, automated packaging was carried out under passive modified atmosphere (p-MAP); three bags per cultivar (stored in the dark, net weight: 500 g *per* bag) were used to make 3 RB of florets as described above. All samples were frozen in liquid nitrogen, ground, and stored at −80°C; samples (20 g) for NMR analysis were weighed without thawing, lyophilized at −50°C for 72 h (FreeZone 1, Labconco, Kansas City, MO, US), and stored at −20°C (Table S3). Three RB were used to provide sufficient material to perform metabolic and transcriptomic analyses; all the measurements were in triplicate.

### Product processing

The plants were harvested to produce shoots (length 25 cm, total weight 15 kg), which were delivered to the company “San Lidano” (www.sanlidano.it) that processed them according to the standards (UNI-EN ISO 9001:2008 for quality; UNI-EN ISO 22005:2007 “100 % Italia” for traceability). Manual selection (cleaning and cutting) produced packable shoots (15–18 cm) and the ratio of bagged to raw weight was 40% (60% waste). Sanitary treatment (sodium hypochlorite 30 mg/L) and washing were carried out at 4–6°C, each batch (5–8 kg) was dried (4400 revolutions per minute) in an automatic electric spin dryer (Nextra compact - Turatti Group, Venice, Italy). Automatic vertical packaging machines (Olimpia 4000 Simotion, Miele, Italy) produced bags filled to a calibrated weight of 500 g ± 2%, which were paced in cartoon boxes, and stored in cold (5 ± 1°C) and dark rooms. To respect the choices for organic products, no gas was flushed (p-MAP, which relies on the product respiration rate and film permeability) and average values (CO_2_ 0.03%, O_2_ 21.8%) were measured by portable gas analyzer (Checkpoint3 Dansensor, Mocon ® Europe) on five randomly selected samples immediately after packaging. The bags characteristics are as follows: anti-fog polypropylene film, size: 645 mm × 380 mm, thickness: 35 μm; density: 910 kg/m^3^ (Masterpack S.p.A., Milan), Oxygen Transmission Rate 1600 cm^3^/m^2^ day atm, water transmission rate: 6 g/m^2^ day.

### Extraction and profiling of metabolite contents

Lyophilized and cold (−20°C) material (50 mg) was added to 1.7 mL of methanol/water (1:1 v/v) into 2.2 mL tubes. The mixture was vortexed for 30 s, centrifuged for 5 min (5000×g), and the supernatant (1.5 mL) was recovered and stored on ice. 1.5 mL of methanol/water (1:1 v/v) was added to the pellet for a second round of extraction as described above. The supernatants were pooled and a final vol of 3 mL was evaporated to dryness under nitrogen stream at room temperature and the residue was dissolved in 0.75 mL of 150 mM phosphate buffer (pH = 7) in deuterated methanol/water (1:1 v/v) added with the internal standard 3-(trimethylsilyl)-propionic-2,2,3,3-d4 acid sodium salt (TSP) at a concentration of 0.5 mM. The NMR spectra of the hydro-soluble compounds were recorded on a Bruker AVANCE III HD 600 NMR spectrometer (27°C, proton frequency 600.13 MHz); the internal standard for the ^1^H spectrum was given by the signal of the TSP methyl groups (δ = 0.00 ppm), each spectrum was acquired by co-addition of 200 transients with a recycle delay of 7 s, using a 45° pulse of 5.8–6.4 μs, 32 K data points. Residual HDO signal was suppressed by pre-saturation. The spectra were further processed using Bruker TOPSPIN software (version 3.6.3), and after Fourier transformation, manual phase and baseline corrections allowed the selection of resonances in the ^1^H NMR spectra (Table S4), which were integrated to calculate the metabolite concentrations, using the integral value of the TSP methyl groups (9H) as a reference for quantification. Metabolite content was expressed as mg/g dry weight. Overall, 25 metabolites were assigned and quantified using the literature data [55], and the following 2D NMR experiments performed as previously reported [56] (^1^H–^1^H total correlation spectroscopy (TOCSY), ^1^H–^13^C heteronuclear single quantum coherence (HSQC) and ^1^H–^13^C heteronuclear multiple bond correlation (HMBC). Briefly, the following key parameters were utilized: the mixing time was 80 ms in TOCSY, the coupling constant *J^1^*_C–H_ was 150 Hz in HSQC, and a delay of 80 ms was used for the evolution of long-range couplings in HMBC.

As for chlorophylls and carotenoids, they were determined by spectrophotometer at 470, 663, and 647 nm and concentrations were expressed as mg/g FW and mg/dm^2^ of leaf area [57]. Mean and standard deviation were calculated using data from six bags (three of cycle1/2021 plus three of cycle2/2022) sampled 1, 4, and 7 days post packaging (DPP). Each sample from each bag consisted of 10 discs from randomly chosen leaves. Storage time (ST) showed significant effects on chlorophyll b decrease in all genotypes, evident at 7 DPP (ca −15 to −30% as mg/g FW in DPP7 vs DPP1). At 4DPP, visual inspection did not reveal significant occurrence of yellowed tissues due to either floret opening or leaf chlorosis. This latter supported by the unvaried content of chlorophyll compared to 1 DPP.

### Transcriptomic analyses

Frost and ground floret tissues derived from the same replicate bulks as those of NMR analyses, and 100 mg was used to isolate total RNA (RNeasy Plant Mini Kit, Qiagen), subsequently digested by 1 DNAse I unit for 5 μg of RNA (ThermoFisher, Waltham, MA, US), further purified by standard procedures (phenol/chloroform treatment and sodium acetate/isopropanol precipitation) and checked for quality (1.2% agarose gel electrophoresis; A260/A280 ratio > 1.8; NanoDrop ND-1000, Thermo Fisher Scientific) and integrity (RIN > 8; BioAnalyzer 2100, Agilent Technologies). The cDNA libraries were synthesized using 1 μg of total RNA by the Universal Plus mRNA-Seq kit (Tecan Genomics), checked with both Qubit 2.0 Fluorometer (Invitrogen) and Bioanalyzer DNA assay (Agilent), and sequenced in paired-end 150 bp mode on NovaSeq 6000 (Illumina). More details on RNA procedures (sampling, etc.) and software used for RNA-seq data analysis were previously described [58] and here used to: a) recover high quality clean reads, which were aligned to the *B. rapa* (cv Chiifu-401-42) reference genome (CAAS_Brap_v3.01, GCF_000309985.2), b) estimate transcript assembly and abundance, c) convert transcript to gene expression level, d) identify DEGs for false discovery rate (FDR) ≤ 0.05 (by Bonferroni-Hochberg method) and absolute log_2_ fold change ≥1; e) assign the KEGG and Gene Onthology (GO) enrichments (p-value cutoff ≤0.05).

### Weighted Gene Coexpression Network Analysis (WGCNA)

Raw counts from 36 datasets (2 genotypes, 3 status, 2 years, 3 replicates) were normalized using the ‘varianceStabilizingTransformation’ tool from the DESeq2 package, and then a filtering step was applied to retain genes above the 85th percentile of gene expression variance (top DEGs). The resulting dataset (5760 genes) was then used for module detection and network construction using the R package WGCNA [59] imposing ‘softPower’ 7, ‘minModuleSize’ 15, and ‘mergeCutHeight’ 0.25 as thresholds. As for the most significant modules *vs* GABA and/or LA content, genes with the highest (top 10%) gene significance, module membership, and intramodular connectivity were selected as genes of interest, and those with a connection strength (edge weight) of 0.2 were visualized with Cytoscape v3.10.1 (cytoscape.org). Finally, genes of interest were used to screen the *B. rapa* collection of cis-regulatory predictions hosted in the PlantRegMap database (plantregmap.gao-lab.org/network.php).

### Statistical analyses

All the statistical analyses were performed within the R environment (v 3.4.3). Variations in the contents of hydrosoluble compounds were subjected to a three-way ANOVA (‘aov’ function of the stats v.3.6.2) with three main factors, namely, genotype, floret status, and year of production, followed by Tukey’s post hoc test (“TukeyHSD” function, p ≤ 0.05). For visual analysis of the data, principal component analysis (PCA) was performed on the mean centered and standardized data and the results were presented as biplots of scores (treatments) and loadings (variables). Pearson correlations of gene vs metabolite and metabolite vs metabolite levels were performed using the “rcorr” function in the Hmisc package and those with p ≤ 0.05 were selected for presentation.

## Supplementary Material

Web_Material_uhae274

## Data Availability

The datasets generated and analysed during the current study has been submitted to the NCBI Sequence Read Archive under the BioProject accession PRJNA112105.
