# Supplementary material for: The molecular pathways leading to GABA and lactic acid accumulation in florets of organic broccoli rabe (Brassica rapa subsp. sylvestris) stored as fresh or as minimally processed product
Source: Hortic Res. 2024 Sep 28;12(1):uhae274. doi: 10.1093/hr/uhae274 (PMC11739617; doi:10.1093/hr/uhae274)
Supplement: Web_Material_uhae274 [file web_material_uhae274.zip › FigureS1.Metabolite correlations.pdf]

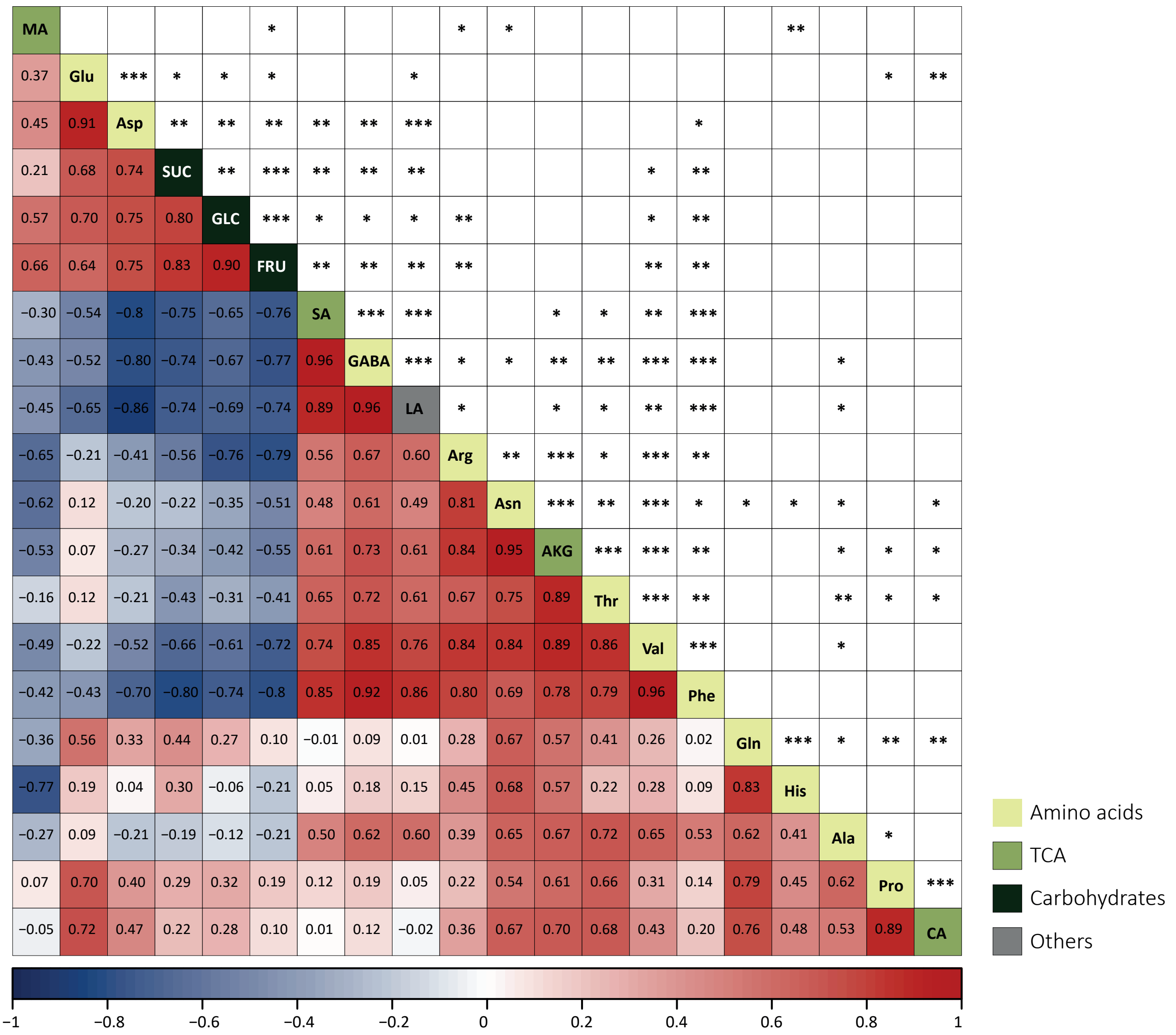

Figure S1. Correlation plot of the Pearson's coefficient (r) among the contents of amino acids (light green), TCAs (green), carbohydrates (dark green) and lactic acid (grey). The correlation coefficients (r) and their significance (asterisks) are arranged in a symmetrically, while a heatmap visually represents the degree of correlation between variables. Red squares represent positive correlations, while blue squares represent negative correlations. *, **, *** = significant at P = 0.05, 0.01 and 0.001, respectively
