# Supplementary material for: The molecular pathways leading to GABA and lactic acid accumulation in florets of organic broccoli rabe (Brassica rapa subsp. sylvestris) stored as fresh or as minimally processed product
Source: Hortic Res. 2024 Sep 28;12(1):uhae274. doi: 10.1093/hr/uhae274 (PMC11739617; doi:10.1093/hr/uhae274)
Supplement: Web_Material_uhae274 [file web_material_uhae274.zip › FigureS2.PCA.pdf]

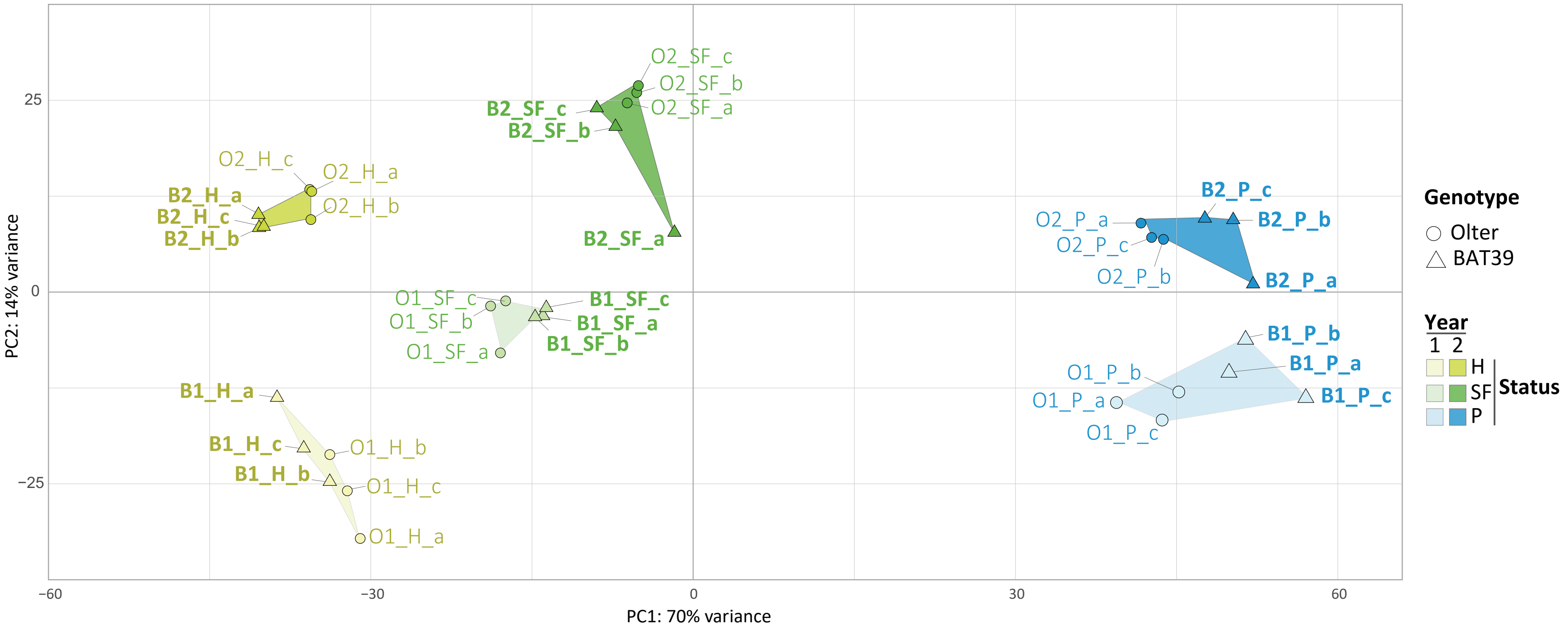

Figure S2. Principal component analysis (PCA) of RNA-seq samples. Color differences indicate the product status (H, harvest; SF, stored as fresh; P, packed) and production cycle (lighter, year 1; darker, year 2); shape differences indicate Olter (circles and light text) and BAT39 (triangles and bold text) cultivars
