# Supplementary material for: The molecular pathways leading to GABA and lactic acid accumulation in florets of organic broccoli rabe (Brassica rapa subsp. sylvestris) stored as fresh or as minimally processed product
Source: Hortic Res. 2024 Sep 28;12(1):uhae274. doi: 10.1093/hr/uhae274 (PMC11739617; doi:10.1093/hr/uhae274)
Supplement: Web_Material_uhae274 [file web_material_uhae274.zip › FigureS3.GO enrichment.pdf]

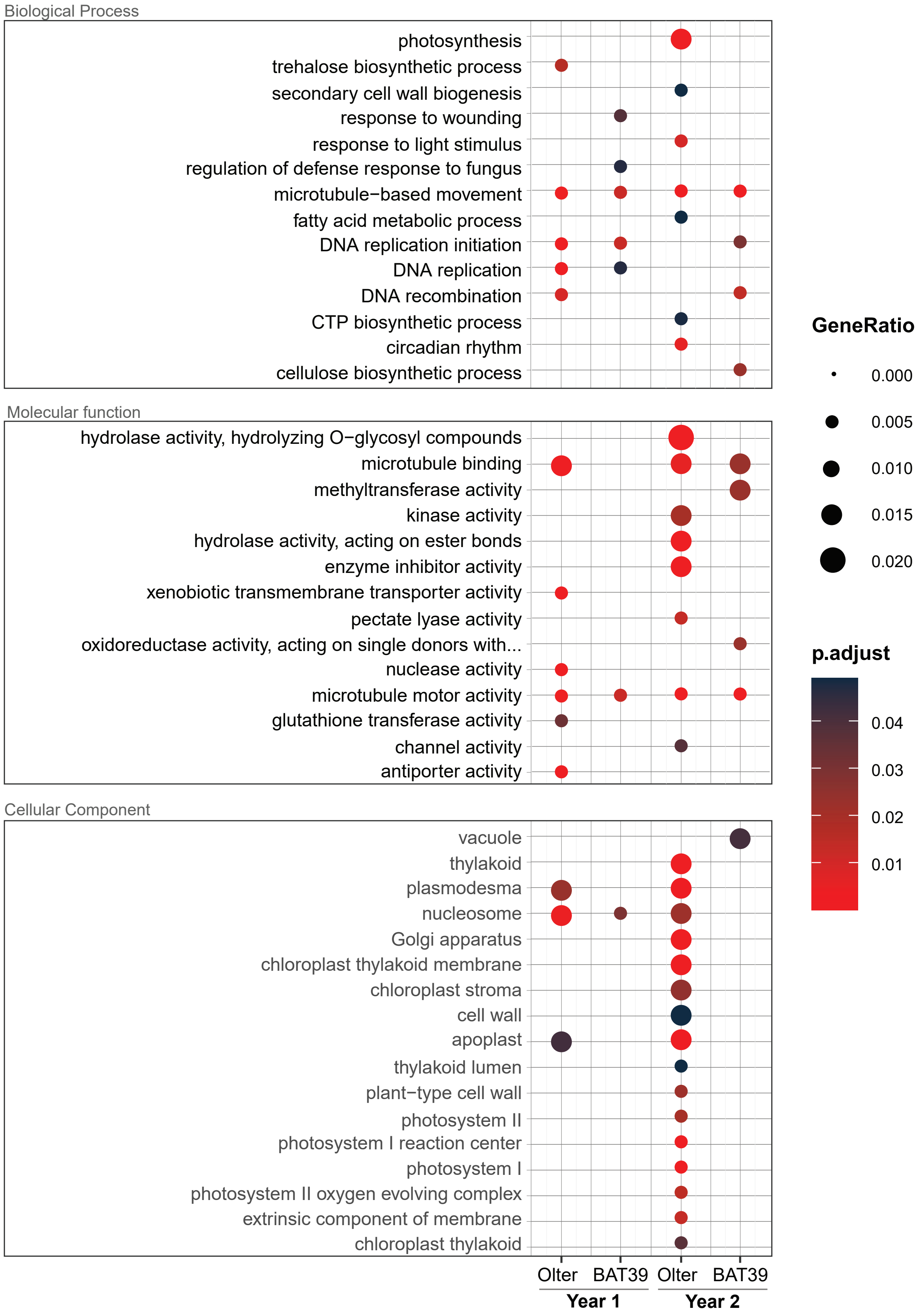

Figure S3. Gene Ontology (GO) enrichment analysis in florets of packed products. The GO terms (y-axis) were grouped into boxes according to their sub-ontologies (biological process, molecular function, and cellular component). The dot size shows the gene ratio in each genotype each year (x-axis). The colour scale shows the adjusted p-adjusted values.
