# Supplementary material for: The molecular pathways leading to GABA and lactic acid accumulation in florets of organic broccoli rabe (Brassica rapa subsp. sylvestris) stored as fresh or as minimally processed product
Source: Hortic Res. 2024 Sep 28;12(1):uhae274. doi: 10.1093/hr/uhae274 (PMC11739617; doi:10.1093/hr/uhae274)
Supplement: Web_Material_uhae274 [file web_material_uhae274.zip › FigureS4.Correlation networkpdf.pdf]

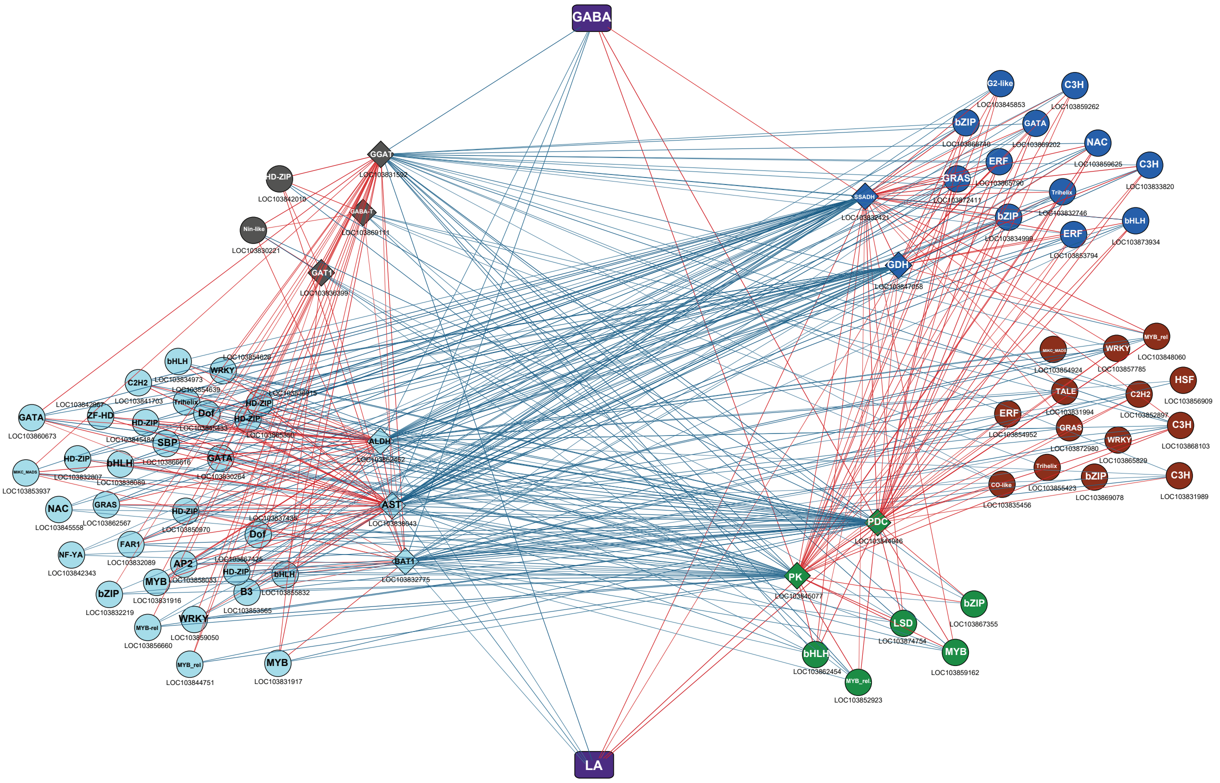

Figure S4. Cytoscape representation of selected genes from green, turquoise, grey60, brown, and blue modules. GABA and lactic acid (LA) are shown as purple boxes. Gene names and annotations (Table S10) are given for transcription factors (circles) and GABA/LA-related genes (diamonds). Positive and negative correlations are indicated by red and blue lines, respectively.
