# Supplementary material for: The molecular pathways leading to GABA and lactic acid accumulation in florets of organic broccoli rabe (Brassica rapa subsp. sylvestris) stored as fresh or as minimally processed product
Source: Hortic Res. 2024 Sep 28;12(1):uhae274. doi: 10.1093/hr/uhae274 (PMC11739617; doi:10.1093/hr/uhae274)
Supplement: Web_Material_uhae274 [file web_material_uhae274.zip › Table S1 - Soil parameters.docx]

**Table S1** Soil characteristics as provided by the Biocaramadre Farm for the year 2020.

| **Soil (USDA)**^1^ | **Sandy loam** |
| --- | --- |
| Clay (<0.002 mm) | 15% |
| Silt (0.05-0.002 mm) | 9% |
| Sand (2-0.05 mm) | 76% |
| Total nitrogen | 0.04% |
| Organic matter | 0.74% |
| P2O5 availability | 71 (mg/kg) |
| K2O exchang. | 211 (mg/kg) |
| E.C. | 0.067 (mS/cm) |
| PH | 6.57 |
| Cat. Ex. Cap. | 12.31 (meq/100g) |

1, Soil classification according to USDA (https://www.nrcs.usda.gov/)
