# Supplementary material for: The molecular pathways leading to GABA and lactic acid accumulation in florets of organic broccoli rabe (Brassica rapa subsp. sylvestris) stored as fresh or as minimally processed product
Source: Hortic Res. 2024 Sep 28;12(1):uhae274. doi: 10.1093/hr/uhae274 (PMC11739617; doi:10.1093/hr/uhae274)
Supplement: Web_Material_uhae274 [file web_material_uhae274.zip › Table S2 - Climate parameters .docx]

**Table S2.** Climate parameters for the two cultivation cycles.

| **Cycle** | **Year** | **Month** | **Tmin °C** | **Tmean °C** | **Tmax °C** | **RH%** | **P (mm)** | **CP (mm)** | **ETr (mm)** |
| --- | --- | --- | --- | --- | --- | --- | --- | --- | --- |
| 1 | 2020 | Nov | 7.09 | 12.40 | 19.75 | 87.88 | 26.20 | 374.00 | 25.95 |
|  | 2020 | Dec | 4.30 | 9.17 | 15.27 | 88.28 | 171.50 | 545.50 | 18.66 |
|  | 2021 | Jan | 1.78 | 7.27 | 13.24 | 86.25 | 129.80 | 129.80 | 22.33 |
|  | 2021 | Feb | 3.68 | 9.71 | 16.30 | 85.62 | 57.70 | 187.50 | 33.40 |
| 2 | 2021 | Nov | 9.47 | 13.64 | 18.84 | 86.78 | 210.90 | 663.90 | 28.97 |
|  | 2021 | Dec | 3.51 | 9.26 | 15.04 | 84.14 | 80.90 | 744.80 | 19.54 |
|  | 2022 | Jan | 1.87 | 7.40 | 14.28 | 80.40 | 18.70 | 18.70 | 22.61 |
|  | 2022 | Feb | 3.11 | 9.64 | 16.51 | 78.30 | 15.00 | 33.70 | 37.85 |
| Mean cycle 1 | | | 4.21 | 9.64 | 16.14 | 87.01 | 96.30 | 309.20 | 25.09 |
| Mean cycle 2 | | | 4.49 | 9.99 | 16.17 | 82.41 | 81.38 | 365.28 | 27.24 |
| Percent change | | | 7% | 4% | 0% | -5% | -15% | 18% | 9% |

Raw data were retrieved from web service of Regione Lazio (<https://siarl.arsial.it/> and <https://www.siarl-lazio.it/E9.asp>) and elaborated. T, temperature; RH, relative humidity; P, precipitation; CP, cumulative precipitation; ETr, reference evapotranspiration. Mean Cyc, mean values for each cycle; the percentage change was calculated by comparing the mean data of cycle 2 *vs* cycle 1.
