# Supplementary material for: The molecular pathways leading to GABA and lactic acid accumulation in florets of organic broccoli rabe (Brassica rapa subsp. sylvestris) stored as fresh or as minimally processed product
Source: Hortic Res. 2024 Sep 28;12(1):uhae274. doi: 10.1093/hr/uhae274 (PMC11739617; doi:10.1093/hr/uhae274)
Supplement: Web_Material_uhae274 [file web_material_uhae274.zip › Table S3 Freeze-dryed data.docx]

**Table S3** Freeze-drying data of florets sampled from “cime di rapa” products in three different conditions for the two cultivation cycles.

| **Sample** | **Status** | **Sample description** | **Year** | **DW/FW** |
| --- | --- | --- | --- | --- |
| BAT39 | H | Harvest | 2021 | 15.3% |
| Olter | H | Harvest | 2021 | 15.2% |
| BAT39 | SF | Stored fresh, 4 days post-harvest | 2021 | 15.2% |
| Olter | SF | Stored fresh, 4 days post-harvest | 2021 | 14.5% |
| BAT39 | P | Stored as bagged, 4 days post-packaging | 2021 | 13.9% |
| Olter | P | Stored as bagged, 4 days post-packaging | 2021 | 14.2% |
| BAT39 | H | Harvest | 2022 | 14.8% |
| Olter | H | Harvest | 2022 | 16.4% |
| BAT39 | SF | Stored fresh, 4 days post-harvest | 2022 | 11.3% |
| Olter | SF | Stored fresh, 4 days post-harvest | 2022 | 15.9% |
| BAT39 | P | Stored as bagged, 4 days post-packaging | 2022 | 14.8% |
| Olter | P | Stored as bagged, 4 days post-packaging | 2022 | 15.2% |
|  |  |  | mean | 14.7% |
|  |  |  | sd | 1.3% |
