# Supplementary material for: The molecular pathways leading to GABA and lactic acid accumulation in florets of organic broccoli rabe (Brassica rapa subsp. sylvestris) stored as fresh or as minimally processed product
Source: Hortic Res. 2024 Sep 28;12(1):uhae274. doi: 10.1093/hr/uhae274 (PMC11739617; doi:10.1093/hr/uhae274)
Supplement: Web_Material_uhae274 [file web_material_uhae274.zip › Table S4 - Characteristic signals in 1H NMR spectra.docx]

**Table S4** Characteristic signals in 1H NMR spectra

| **Metabolite^a^** | **Group** | **Integral range** | |
| --- | --- | --- | --- |
|  |  | **From (ppm)** | **To (ppm)** |
| Ile | γCH_3_ | 1.044 | 1.032 |
| Val | γCH_3_ | 1.082 | 1.055 |
| Thr | γCH_3_ | 1.361 | 1.347 |
| Ala | CH_3_ | 1.512 | 1.473 |
| Arg | γCH_2_ | 1.714 | 1.703 |
| Pro | γCH_2_ | 2.014 | 1.967 |
| Gln | γCH_2_ | 2.487 | 2.437 |
| Glu | γCH_2_ | 2.402 | 2.389 |
| Asp | β-CH_2_ | 2.804 | 2.777 |
| Asn | β-CH_2_ | 2.863 | 2.834 |
| Phe | 2,6-CH | 7.422 | 7.380 |
| His | 2-CH (ring) | 7.799 | 7.786 |
| GABA | αCH_2_ | 2.318 | 2.297 |
| Succinic acid (SA) | α,β-CH_2_ | 2.410 | 2.405 |
| Citric acid (CA) | α,γ-CH | 2.536 | 2.512 |
| Malic acid (MA) | αCH | 4.297 | 4.251 |
| α-Ketoglutaric acid (AKG) | β-CH_2_ | 2.541 | 2.536 |
| α-Glucose (GLC)^b^ | CH-1 | 5.211 | 5.193 |
| β-Glucose (GLC)^b^ | CH-1 | 4.616 | 4.581 |
| Fructose (FRU) | CH-6 (β-fructopyranose) | 4.032 | 4.015 |
| Sucrose (SUC) | CH-1 (glucose ring) | 5.427 | 5.401 |
| Lactic acid (LA) | CH_3_ | 1.334 | 1.322 |
| Quercetin (QCT) derivative | 2’-CH | 7.864 | 7.842 |
| Ethanolamine (ETA) | CH_2_NH_2_ | 3.140 | 3.108 |
| Choline (CHO) | N(CH_3_)_3_ | 3.230 | 3.214 |
| Methiin (MET) | CH_3_(S=O) | 2.819 | 2.810 |

^a^ Abbreviation is in parenthesis;

^b^ GLC content was the sum of α and β isomers
