# Supplementary material for: The molecular pathways leading to GABA and lactic acid accumulation in florets of organic broccoli rabe (Brassica rapa subsp. sylvestris) stored as fresh or as minimally processed product
Source: Hortic Res. 2024 Sep 28;12(1):uhae274. doi: 10.1093/hr/uhae274 (PMC11739617; doi:10.1093/hr/uhae274)
Supplement: Web_Material_uhae274 [file web_material_uhae274.zip › Table S5 - Variations of the hydrosoluble compound means in florets.docx]

**Table** **S5** Mean values of the hydrosoluble compound abundances (mg/g DW) in florets and relative changes vs freshly harvested products.

|  | Year 1 | | | | | | | | | | | | | | Year 2 | | | | | | | | | | | | | |
| --- | --- | --- | --- | --- | --- | --- | --- | --- | --- | --- | --- | --- | --- | --- | --- | --- | --- | --- | --- | --- | --- | --- | --- | --- | --- | --- | --- | --- |
|  | Mean values | | | | | | Fold changes | | | | | | | | Mean values | | | | | | Fold changes | | | | | | | |
|  | BAT39 | | | Olter | | | BAT39 | | | | Olter | | | | BAT39 | | | Olter | | | BAT39 | | | | Olter | | | |
| **Compound** | **H** | **SF** | **P** | **H** | **SF** | **P** | **SFvsH** | | **PvsH** | | **SFvsH** | | **PvsH** | | **H** | **SF** | **P** | **H** | **SF** | **P** | **SFvsH** | | **PvsH** | | **SFvsH** | | **PvsH** | |
| Ile | 0.13 | 0.39 | 0.65 | 0.18 | 0.28 | 0.55 | 194% | ▲ | 388% | ▲ | 57% | ▲ | 212% | ▲ | 0.16 | 0.2 | 0.47 | 0.11 | 0.32 | 0.38 | 25% |  | 185% | ▲ | 189% | ▲ | 236% | ▲ |
| Val | 0.29 | 0.85 | 1.42 | 0.39 | 0.66 | 1.29 | 192% | ▲ | 390% | ▲ | 69% | ▲ | 229% | ▲ | 0.4 | 0.39 | 0.94 | 0.27 | 0.7 | 0.8 | -2% |  | 134% | ▲ | 158% | ▲ | 196% | ▲ |
| Thr | 0.41 | 0.71 | 0.84 | 0.52 | 0.51 | 0.74 | 72% | ▲ | 105% | ▲ | -1% |  | 43% |  | 0.27 | 0.37 | 0.58 | 0.2 | 0.37 | 0.53 | 38% |  | 115% | ▲ | 85% | ▲ | 166% | ▲ |
| Ala | 0.82 | 1.34 | 1.98 | 0.94 | 1.04 | 1.16 | 63% | ▲ | 141% | ▲ | 10% |  | 23% |  | 1.31 | 0.29 | 1.56 | 0.3 | 0.39 | 0.86 | -78% | ▼ | 19% |  | 31% |  | 187% | ▲ |
| Arg | 0.96 | 1.82 | 2.59 | 0.83 | 1.32 | 1.81 | 89% | ▲ | 169% | ▲ | 59% | ▲ | 118% | ▲ | 0.72 | 0.8 | 1.46 | 1.37 | 1.7 | 1.69 | 12% |  | 104% | ▲ | 24% |  | 23% |  |
| Pro | 4.61 | 4.83 | 4.51 | 4.99 | 3.95 | 4.14 | 5% |  | -2% |  | -21% |  | -17% |  | 1.9 | 1.12 | 2.25 | 1.49 | 1.54 | 1.63 | -41% |  | 19% |  | 3% |  | 9% |  |
| Gln | 13.46 | 20.34 | 13.92 | 17.32 | 13.15 | 13.59 | 51% | ▲ | 3% |  | -24% |  | -22% |  | 13.11 | 2.99 | 9.92 | 9.63 | 9.54 | 9.55 | -77% | ▼ | -24% |  | -1% |  | -1% |  |
| Glu | 6.49 | 8.6 | 2.86 | 7.56 | 7.53 | 3.64 | 33% |  | -56% | ▼ | 0% |  | -52% | ▼ | 3.61 | 2.36 | 0.78 | 2.22 | 3.23 | 0.99 | -34% |  | -78% | ▼ | 46% |  | -56% | ▼ |
| Asp | 3.08 | 4.96 | 0.7 | 3.75 | 4.31 | 1.06 | 61% | ▲ | -77% | ▼ | 15% |  | -72% | ▼ | 2.06 | 1.92 | 0.29 | 2.03 | 1.81 | 0.52 | -7% |  | -86% | ▼ | -11% |  | -74% | ▼ |
| Asn | 1.18 | 2.44 | 3.18 | 1.78 | 1.84 | 2.77 | 108% | ▲ | 170% | ▲ | 4% |  | 56% | ▲ | 1.36 | 0.59 | 1.82 | 1.2 | 2.23 | 1.85 | -57% | ▼ | 34% |  | 86% | ▲ | 54% | ▲ |
| Phe | 0.06 | 0.26 | 0.68 | 0.07 | 0.16 | 0.57 | 375% | ▲ | 1133% | ▲ | 149% | ▲ | 770% | ▲ | 0.07 | 0.19 | 0.45 | 0.05 | 0.25 | 0.38 | 181% | ▲ | 550% | ▲ | 384% | ▲ | 630% | ▲ |
| His | 0.69 | 0.77 | 0.69 | 0.72 | 0.76 | 0.7 | 11% |  | 0% |  | 6% |  | -2% |  | 0.64 | 0.13 | 0.71 | 0.73 | 0.76 | 0.74 | -80% | ▼ | 10% |  | 5% |  | 2% |  |
| GABA | 0.23 | 0.24 | 8.11 | 0.23 | 0.21 | 5.58 | 4% |  | 3478% | ▲ | -8% |  | 2309% | ▲ | 0.13 | 0.15 | 5.14 | 0.09 | 0.18 | 2.78 | 15% |  | 3745% | ▲ | 93% | ▲ | 2837% | ▲ |
| **TOT.AA** | **32.41** | **47.55** | **42.13** | **39.26** | **35.73** | **37.59** | **47%** |  | **30%** |  | **-9%** |  | **-4%** |  | **25.73** | **11.52** | **26.37** | **19.69** | **23.02** | **22.69** | **-55%** |  | **2%** |  | **17%** |  | **15%** |  |
| SA | 0.12 | 0.05 | 0.91 | 0.19 | 0.04 | 0.82 | -61% | ▼ | 664% | ▲ | -79% | ▼ | 329% | ▲ | 0.1 | 0.2 | 0.53 | 0.1 | 0.03 | 0.68 | 89% | ▲ | 411% | ▲ | -65% | ▼ | 618% | ▲ |
| CA | 7.82 | 12.87 | 9.8 | 11.05 | 11.66 | 10.56 | 65% | ▲ | 25% |  | 6% |  | -4% |  | 6.03 | 5.1 | 6.9 | 5.71 | 7.36 | 5.76 | -15% |  | 14% |  | 29% |  | 1% |  |
| MA | 5.19 | 3.97 | 2.93 | 6.73 | 3.05 | 2.98 | -23% |  | -43% | ▼ | -55% | ▼ | -56% | ▼ | 3.86 | 12.31 | 2.28 | 2.81 | 1.9 | 1.67 | 219% | ▲ | -41% | ▼ | -32% |  | -40% | ▼ |
| AKG | 0.49 | 0.92 | 1.33 | 0.65 | 0.74 | 1.15 | 88% | ▲ | 171% | ▲ | 13% |  | 76% | ▲ | 0.29 | 0.17 | 0.8 | 0.34 | 0.72 | 0.75 | -40% |  | 179% | ▲ | 112% | ▲ | 124% | ▲ |
| **TOT.TCA** | **13.61** | **17.81** | **14.97** | **18.63** | **15.5** | **15.51** | **31%** |  | **10%** |  | **-17%** |  | **-17%** |  | **10.28** | **17.79** | **10.51** | **8.94** | **10** | **0.38** | **73%** |  | **2%** |  | **12%** |  | **-96%** |  |
| GLC | 6.95 | 6.03 | 1.79 | 12.61 | 5.92 | 4.44 | -13% |  | -74% | ▼ | -53% | ▼ | -65% | ▼ | 8.05 | 5.02 | 3.57 | 4.04 | 3.97 | 1.96 | -38% |  | -56% | ▼ | -2% |  | -52% | ▼ |
| FRU | 11.53 | 7.51 | 2.57 | 13.67 | 6.35 | 2.77 | -35% |  | -78% | ▼ | -54% | ▼ | -80% | ▼ | 9.49 | 8.05 | 4.26 | 4.28 | 5.94 | 2.31 | -15% |  | -55% | ▼ | 39% |  | -46% |  |
| SUC | 13.56 | 5.62 | 0.81 | 13.24 | 4.97 | 1.37 | -59% | ▼ | -94% | ▼ | -62% | ▼ | -90% | ▼ | 7.22 | 1.4 | 1.64 | 3.9 | 6.52 | 1.64 | -81% | ▼ | -77% | ▼ | 67% | ▲ | -58% | ▼ |
| **TOT.CAR** | **32.03** | **19.17** | **5.17** | **39.52** | **17.23** | **8.58** | **-40%** |  | **-84%** |  | **-56%** |  | **-78%** |  | **24.77** | **14.48** | **9.47** | **12.23** | **16.43** | **5.91** | **-42%** |  | **-62%** |  | **34%** |  | **-52%** |  |
| LA | 0.00 | 0.00 | 1.55 | 0.00 | 0.00 | 0.78 | 0% |  | 154800% | ▲ | 0% |  | 77533% | ▲ | 0.01 | 0.03 | 1.89 | 0.01 | 0.01 | 0.86 | 148% | ▲ | 18171% | ▲ | 113% | ▲ | 15950% | ▲ |
| QCT | 0.28 | 0.35 | 0.81 | 0.16 | 0.16 | 0.42 | 22% |  | 186% | ▲ | 2% |  | 171% | ▲ | 0.54 | 1.36 | 1.35 | 0.58 | 0.56 | 0.78 | 152% | ▲ | 150% | ▲ | -4% |  | 34% |  |
| ETA | 0.61 | 0.77 | 0.78 | 0.78 | 0.64 | 0.71 | 26% |  | 27% |  | -17% |  | -9% |  | 0.63 | 0.27 | 0.64 | 0.53 | 0.56 | 0.53 | -57% | ▼ | 2% |  | 7% |  | 1% |  |
| CHO | 1.99 | 2.35 | 2.44 | 2.51 | 2.07 | 2.32 | 19% |  | 23% |  | -18% |  | -8% |  | 2.06 | 0.82 | 1.97 | 1.84 | 1.83 | 1.64 | -60% | ▼ | -4% |  | 0% |  | -11% |  |
| MET | 3.02 | 1.77 | 2.45 | 3.92 | 4.96 | 3.89 | -41% |  | -19% |  | 27% |  | -1% |  | 4.92 | 1.77 | 3.96 | 5.42 | 4.96 | 3.47 | -64% | ▼ | -19% |  | -8% |  | -36% |  |

Florets at harvest, H; stored as fresh, SF; packaged, P (see materials and methods for further details). SF vs H (and P vs H) indicate the comparison between florets of SF (or P) vs those from H. Relative changes (RC) of each metabolite were determined by formula RC = [(SF-H)/H]. Arrowheads are indicated for positive values higher than 50% (red) or negative lower than -50% (blue). **Amino acids:** Ile, Isoleucine; Val, Valine; Thr, Threonine; Ala, Alanine; Arg, Arginine; Pro, Proline; Gln, Glutamine; Glu, Glutamic acid; Asp, Aspartic acid; Asn, Asparagine; Phe, Phenylalanine; His, Histidine; GABA, gamma-Aminobutyric acid; TOT.AA, total amino acids. **Tricarboxylic acids:** SA, Succinic acid; CA, Citric acid; MA, Malic acid; AKG, a-Ketoglutaric acid; TOT.TCA, Total Tricarboxylic acids; **Carbohydrates**: GLC, Glucose; FRU, Fructose; SUC, Sucrose; TOT.CAR, total carbohydrates. **Other compounds**: LA, Lactic acid; QCT, Quercitin; ETA, Ethanolamine; CHO, Choline; MET, Methiin.
