# Supplementary material for: The molecular pathways leading to GABA and lactic acid accumulation in florets of organic broccoli rabe (Brassica rapa subsp. sylvestris) stored as fresh or as minimally processed product
Source: Hortic Res. 2024 Sep 28;12(1):uhae274. doi: 10.1093/hr/uhae274 (PMC11739617; doi:10.1093/hr/uhae274)
Supplement: Web_Material_uhae274 [file web_material_uhae274.zip › Table S7 - Variations of the hydrosoluble compound means in leaves.docx]

### **Table S7**. Mean values of the hydrosoluble compound abundances (mg/g DW) in leaves and relative changes vs freshly harvested products.

|  | Year 1 | | | | | | | | | | | | | | Year 2 | | | | | | | | | | | | | |
| --- | --- | --- | --- | --- | --- | --- | --- | --- | --- | --- | --- | --- | --- | --- | --- | --- | --- | --- | --- | --- | --- | --- | --- | --- | --- | --- | --- | --- |
|  | Mean values | | | | | | Relative changes | | | | | | | | Mean values | | | | | | Relative changes | | | | | | | |
|  | BAT39 | | | Olter | | | BAT39 | | | | Olter | | | | BAT39 | | | Olter | | | BAT39 | | | | Olter | | | |
| **Compounds** | **H** | **SF** | **P** | **H** | **SF** | **P** | **SFvsH** | | **PvsH** | | **SFvsH** | | **PvsH** | | **H** | **SF** | **P** | **H** | **SF** | **P** | **SFvsH** | | **PvsH** | | **SFvsH** | | **PvsH** | |
| Ile | 0.13 | 0.27 | 0.52 | 0.11 | 0.32 | 0.48 | 108% | ▲ | 300% | ▲ | 191% | ▲ | 336% | ▲ | 0.07 | 0.20 | 0.25 | 0.08 | 0.25 | 0.24 | 186% | ▲ | 257% | ▲ | 213% | ▲ | 200% | ▲ |
| Val | 0.20 | 0.57 | 0.95 | 0.18 | 0.59 | 0.91 | 185% | ▲ | 375% | ▲ | 228% | ▲ | 406% | ▲ | 0.08 | 0.39 | 0.50 | 0.08 | 0.49 | 0.48 | 388% | ▲ | 525% | ▲ | 513% | ▲ | 500% | ▲ |
| Thr | 0.85 | 1.00 | 0.94 | 0.72 | 0.91 | 0.83 | 18% |  | 11% |  | 26% |  | 15% |  | 0.17 | 0.39 | 0.42 | 0.20 | 0.48 | 0.44 | 129% | ▲ | 147% | ▲ | 140% | ▲ | 120% | ▲ |
| Ala | 0.65 | 0.73 | 1.98 | 0.53 | 0.83 | 0.84 | 12% |  | 205% | ▲ | 57% | ▲ | 58% | ▲ | 0.19 | 0.28 | 1.05 | 0.18 | 0.33 | 0.90 | 47% |  | 453% | ▲ | 83% | ▲ | 400% | ▲ |
| Arg | 0.86 | 1.18 | 1.68 | 0.85 | 1.57 | 1.96 | 37% |  | 95% | ▲ | 85% | ▲ | 131% | ▲ | 0.39 | 0.75 | 0.87 | 0.38 | 0.85 | 1.06 | 92% | ▲ | 123% | ▲ | 124% | ▲ | 179% | ▲ |
| Pro | 3.53 | 2.11 | 2.45 | 3.51 | 3.97 | 2.75 | -40% |  | -31% |  | 13% |  | -22% |  | 1.21 | 1.04 | 1.14 | 1.02 | 1.00 | 1.48 | -14% |  | -6% |  | -2% |  | 45% |  |
| Gln | 4.72 | 3.17 | 4.84 | 4.25 | 4.55 | 7.00 | -33% |  | 3% |  | 7% |  | 65% | ▲ | 3.79 | 3.01 | 2.59 | 2.08 | 2.52 | 4.22 | -21% |  | -32% |  | 21% |  | 103% | ▲ |
| Glu | 7.39 | 4.80 | 1.73 | 6.29 | 5.23 | 2.64 | -35% |  | -77% | ▼ | -17% |  | -58% | ▼ | 2.13 | 2.39 | 0.45 | 1.65 | 2.70 | 0.75 | 12% |  | -79% | ▼ | 64% | ▲ | -55% | ▼ |
| Asp | 4.12 | 4.15 | 0.45 | 3.74 | 4.76 | 0.69 | 1% |  | -89% | ▼ | 27% |  | -82% | ▼ | 1.30 | 1.90 | 0.09 | 1.21 | 1.72 | 0.24 | 46% |  | -93% | ▼ | 42% |  | -80% | ▼ |
| Asn | 0.51 | 0.59 | 0.94 | 0.63 | 0.97 | 1.40 | 16% |  | 84% | ▲ | 54% | ▲ | 122% | ▲ | 0.40 | 0.60 | 0.70 | 0.35 | 0.52 | 0.85 | 50% |  | 75% | ▲ | 49% |  | 143% | ▲ |
| Phe | 0.13 | 0.29 | 0.52 | 0.10 | 0.31 | 0.50 | 123% | ▲ | 300% | ▲ | 210% | ▲ | 400% | ▲ | 0.03 | 0.19 | 0.27 | 0.03 | 0.22 | 0.25 | 533% | ▲ | 800% | ▲ | 633% | ▲ | 733% | ▲ |
| His | 0.12 | 0.18 | 0.29 | 0.10 | 0.25 | 0.33 | 50% |  | 142% | ▲ | 150% | ▲ | 230% | ▲ | 0.05 | 0.17 | 0.21 | 0.05 | 0.16 | 0.22 | 240% | ▲ | 320% | ▲ | 220% | ▲ | 340% | ▲ |
| GABA | 0.28 | 0.10 | 7.98 | 0.13 | 0.10 | 4.83 | -64% | ▼ | 2750% | ▲ | -23% |  | 3615% | ▲ | 0.03 | 0.13 | 3.15 | 0.05 | 0.12 | 2.64 | 333% | ▲ | 10400% | ▲ | 140% | ▲ | 5180% | ▲ |
| **TOT.AA** | **23.50** | **19.14** | **25.27** | **21.15** | **24.36** | **25.14** | **-19%** |  | **8%** |  | **15%** |  | **19%** |  | **9.82** | **11.44** | **11.69** | **7.35** | **11.37** | **13.77** | **16%** |  | **19%** |  | **55%** |  | **87%** |  |
| SA | 0.56 | 0.17 | 2.31 | 0.46 | 0.14 | 1.89 | -70% | ▼ | 313% | ▲ | -70% | ▼ | 311% | ▲ | 0.73 | 0.18 | 1.10 | 0.66 | 0.10 | 1.08 | -75% | ▼ | 51% | ▲ | -85% | ▼ | 64% | ▲ |
| CA | 5.52 | 7.65 | 7.16 | 5.10 | 9.56 | 8.52 | 39% |  | 30% |  | 87% | ▲ | 67% | ▲ | 2.38 | 4.92 | 3.56 | 2.92 | 5.85 | 4.40 | 107% | ▲ | 50% |  | 100% | ▲ | 51% | ▲ |
| MA | 29.62 | 20.35 | 20.83 | 24.88 | 20.25 | 15.13 | -31% |  | -30% |  | -19% |  | -39% |  | 15.56 | 11.99 | 9.49 | 11.71 | 12.87 | 9.26 | -23% |  | -39% |  | 10% |  | -21% |  |
| AKG | 0.18 | 0.23 | 0.43 | 0.17 | 0.30 | 0.47 | 28% |  | 139% | ▲ | 76% | ▲ | 176% | ▲ | 0.08 | 0.17 | 0.20 | 0.10 | 0.20 | 0.25 | 113% | ▲ | 150% | ▲ | 100% | ▲ | 150% | ▲ |
| **TOT.TCA** | **35.87** | **28.39** | **30.73** | **30.60** | **30.26** | **26.02** | **-21%** |  | **-14%** |  | **-1%** |  | **-15%** |  | **18.74** | **17.26** | **14.35** | **15.39** | **19.01** | **14.98** | **-8%** |  | **-23%** |  | **24%** |  | **-3%** |  |
| GLC | 12.55 | 7.08 | 7.44 | 11.29 | 10.46 | 7.76 | -44% |  | -41% |  | -7% |  | -31% |  | 10.91 | 5.24 | 11.26 | 8.16 | 11.77 | 8.72 | -52% | ▼ | 3% |  | 44% |  | 7% |  |
| FRU | 31.59 | 17.79 | 10.28 | 29.34 | 23.89 | 13.31 | -44% |  | -67% | ▼ | -19% |  | -55% | ▼ | 20.17 | 8.81 | 15.38 | 15.66 | 17.79 | 13.00 | -56% | ▼ | -24% |  | 14% |  | -17% |  |
| SUC | 15.73 | 1.56 | 1.36 | 12.56 | 2.81 | 1.56 | -90% | ▼ | -91% | ▼ | -78% | ▼ | -88% | ▼ | 3.71 | 1.56 | 1.63 | 3.02 | 1.82 | 1.54 | -58% | ▼ | -56% | ▼ | -40% |  | -49% |  |
| **TOT.CAR** | **59.86** | **26.44** | **19.08** | **53.19** | **37.16** | **22.63** | **-56%** |  | **-68%** |  | **-30%** |  | **-57%** |  | **34.79** | **15.61** | **28.28** | **26.83** | **31.39** | **23.26** | **-55%** |  | **-19%** |  | **17%** |  | **-13%** |  |
| LA | 0.00 | 0.00 | 0.52 | 0.00 | 0.00 | 0.09 | 0% |  | 51900% | ▲ | 0% |  | 8900% | ▲ | 0.02 | 0.03 | 0.65 | 0.01 | 0.09 | 0.31 | 50% |  | 3150% | ▲ | 800% | ▲ | 3000% | ▲ |
| QCT | 0.78 | 0.79 | 1.05 | 0.58 | 0.72 | 0.66 | 1% |  | 35% |  | 24% |  | 14% |  | 1.20 | 1.34 | 1.20 | 1.59 | 1.47 | 1.20 | 12% |  | 0% |  | -8% |  | -25% |  |
| ETA | 0.44 | 0.35 | 0.48 | 0.42 | 0.44 | 0.48 | -20% |  | 9% |  | 5% |  | 14% |  | 0.28 | 0.28 | 0.35 | 0.24 | 0.29 | 0.36 | 0% |  | 25% |  | 21% |  | 50% |  |
| CHO | 1.27 | 0.99 | 1.22 | 1.13 | 1.22 | 1.19 | -22% |  | -4% |  | 8% |  | 5% |  | 1.02 | 0.79 | 1.12 | 0.87 | 1.01 | 1.09 | -23% |  | 10% |  | 16% |  | 25% |  |
| MET | 1.29 | 0.97 | 0.89 | 1.39 | 1.65 | 2.33 | -25% |  | -31% |  | 19% |  | 68% | ▲ | 2.09 | 1.75 | 1.77 | 1.78 | 1.81 | 1.69 | -16% |  | -15% |  | 2% |  | -5% |  |

Leaves at harvest, H; stored as fresh, SF; packaged, P (see materials and methods for further details). SF vs H (and P vs H) indicate the comparison between florets of SF (or P) vs those from H. Relative changes (RC) of each metabolite were determined by formula RC = [(SF-H)/H]. Arrowheads are indicated for positive values higher than 50% (red) or negative lower than -50% (blue). **Amino acids:** Ile, Isoleucine; Val, Valine; Thr, Threonine; Ala, Alanine; Arg, Arginine; Pro, Proline; Gln, Glutamine; Glu, Glutamic acid; Asp, Aspartic acid; Asn, Asparagine; Phe, Phenylalanine; His, Histidine; GABA, gamma-Aminobutyric acid; TOT.AA, total amino acids. **Tricarboxylic acids:** SA, Succinic acid; CA, Citric acid; MA, Malic acid; AKG, a-Ketoglutaric acid; TOT.TCA, Total Tricarboxylic acids; **Carbohydrates**: GLC, Glucose; FRU, Fructose; SUC, Sucrose; TOT.CAR, total carbohydrates. **Other compounds**: LA, Lactic acid; QCT, Quercitin; ETA, Ethanolamine; CHO, Choline; MET, Methiin.
