# Supplementary material for: The molecular pathways leading to GABA and lactic acid accumulation in florets of organic broccoli rabe (Brassica rapa subsp. sylvestris) stored as fresh or as minimally processed product
Source: Hortic Res. 2024 Sep 28;12(1):uhae274. doi: 10.1093/hr/uhae274 (PMC11739617; doi:10.1093/hr/uhae274)
Supplement: Web_Material_uhae274 [file web_material_uhae274.zip › Table S8 - Gas composition in packaged produts_revised.docx]

**Table S8** Gas variation in packaged product

|  | **CO_2_%** | | **O_2_%** | |
| --- | --- | --- | --- | --- |
| **DPP** | **BAT39** | **OLTER** | **BAT39** | **OLTER** |
| 0 | 0.21±0.03 | 0.20±0.03 | 21.23±3.19 | 21.17±3.18 |
| 4 | 9.01±1.35 | 8.51±1.28 | 11.11±1.67 | 12.05±1.81 |
| 7 | 16.66±1.33 | 14.80±1.33 | 5.76±1.15 | 7.02±1.40 |

Measurements were made with a portable gas analyzer (Checkpoint3 Dansensor, Mocon ® Europe); mean and standard deviation were calculated using data from 6 bags (3 of cycle1/2021 *plus* 3 of cycle2/2022) per sampling time, indicated as day post packaging (DPP). 4 DPP is the date of most likely consumption and targeted for all analyses; testing days beyond the sell-by date (7 DPP) was beyond the scope of the work (see also section sampling methods and criteria). Carbon dioxide content is known to exponentially increase within few days from packaging in broccoli (reference from the article) and this trend was confirmed at 4DPP and was independent of the genotype.
