# Supplementary material for: The molecular pathways leading to GABA and lactic acid accumulation in florets of organic broccoli rabe (Brassica rapa subsp. sylvestris) stored as fresh or as minimally processed product
Source: Hortic Res. 2024 Sep 28;12(1):uhae274. doi: 10.1093/hr/uhae274 (PMC11739617; doi:10.1093/hr/uhae274)
Supplement: Web_Material_uhae274 [file web_material_uhae274.zip › Table S9 - Pigment content variations in packaged products.docx]

**Table S9.** Chlorophyll and carotenoid content variation in packaged product

| **Genotype** | **Storage**  **DPP** | **Chla**  **(mg**/**g FW)** | **Chlb**  **(mg**/**g FW)** | **Chla**  **(mg**/**dm)** | **Chlb**  **(mg**/**dm)** | **CAR**  **(mg**/**g FW)** | **CAR**  **(mg**/**dm)** |
| --- | --- | --- | --- | --- | --- | --- | --- |
| BAT39 | 1 | 1.27±0.23 | 0.70±0.21 | 2.26±0.478ab | 1.25±0.41 | 0.33±0.06 | 0.59±0.12 |
|  | 4 | 1.18±0.17 | 0.63±0.04 | 2.18±0.32ab | 1.18±0.11 | 0.32±0.07 | 0.61±0.19 |
|  | 7 | 1.46±0.22 | 0.60±0.22 | 2.44±0.28ab | 0.99±0.32 | 0.38±0.04 | 0.64±0.07 |
| Olter | 1 | 1.45±0.15 | 0.73±0.15 | 2.86±0.51a | 1.45±0.4 | 0.34±0.02 | 0.68±0.07 |
|  | 4 | 1.41±0.11 | 0.86±0.13 | 2.47±0.21ab | 1.51±0.16 | 0.28±0.04 | 0.49±0.08 |
|  | 7 | 1.11±0.21 | 0.46±0.21 | 1.89±0.206b | 0.77±0.29 | 0.33±0.07 | 0.55±0.04 |
| Sign. | G | ns | ns | ns | ns | * | ns |
|  | T | ns | ** | ns | *** | ns | ns |
|  | GxT | ns | ns | * | ns | ns | ns |

The photosynthetic pigments were extracted (2 ml 80% acetone by centrifugation at 4 °C and 10000 rpm for 4 min; 0.5 ml of the supernatant was removed from the initial extract and stored at 4 °C in the dark for the analyses. Chlorophylls and carotenoids were determined spectrophotometrycally^58^ at 470, 663 and 647 nm and concentration were expressed in mg vs fresh weight (mg/g FW) and leaf area (mg/dm^2^). Mean and standard deviation were calculated using data from 6 bags (3 of cycle1/2021 plus 3 of cycle2/2022) sampled 1-, 4- and 7-days post packaging (DPP). Each sample from each bag consisted of 10 discs from randomly chosen leaves. Storage time (ST) showed significant effects on chlorophyll b decrease in all genotypes, evident at 7 DPP (ca -15 to -30% as mg/g FW in DPP7 vs DPP1). At 4DPP, visual inspection did not reveal significant occurrence of yellowed tissues due to either floret opening or leaf chlorosis, this latter supported by the unvaried content of chlorophyll compared to 1 DPP.
